# The Codacs^™^ Direct Acoustic Cochlear Implant Actuator: Exploring Alternative Stimulation Sites and Their Stimulation Efficiency

**DOI:** 10.1371/journal.pone.0119601

**Published:** 2015-03-18

**Authors:** Martin Grossöhmichen, Rolf Salcher, Hans-Heinrich Kreipe, Thomas Lenarz, Hannes Maier

**Affiliations:** 1 Department of Otolaryngology and Institute of Audioneurotechnology (VIANNA), Hannover Medical School, Hannover, Germany; 2 Institute for Pathology, Hannover Medical School, Hannover, Germany; University of Zurich, SWITZERLAND

## Abstract

This work assesses the efficiency of the Codacs system actuator (Cochlear Ltd., Sydney Australia) in different inner ear stimulation modalities. Originally the actuator was intended for direct perilymph stimulation after stapedotomy using a piston prosthesis. A possible alternative application is the stimulation of middle ear structures or the round window (RW). Here the perilymph stimulation with a K-piston through a stapes footplate (SFP) fenestration (N = 10) as well as stimulation of the stapes head (SH) with a Bell prosthesis (N = 9), SFP stimulation with an Omega/Aerial prosthesis (N = 8) and reverse RW stimulation (N = 10) were performed in cadaveric human temporal bones (TBs). Codacs actuator output is expressed as equivalent sound pressure level (eq. SPL) using RW and SFP displacement responses, measured by Laser Doppler velocimetry as reference. The axial actuator coupling force in stimulation of stapes and RW was adjusted to ~ 5 mN. The Bell prosthesis and Omega/Aerial prosthesis stimulation generated similar mean eq. SPLs (Bell: 127.5–141.8 eq. dB SPL; Omega/Aerial: 123.6–143.9 eq. dB SPL), being significantly more efficient than K-piston perilymph stimulation (108.6–131.6 eq. dB SPL) and RW stimulation (108.3–128.2 eq. dB SPL). Our results demonstrate that SH, SFP and RW are adequate alternative stimulation sites for the Codacs actuator using coupling prostheses and an axial coupling force of ~ 5 mN. Based on the eq. SPLs, all investigated methods were adequate for *in vivo* hearing aid applications, provided that experimental conditions including constant coupling force will be implemented.

## Introduction

**Fig 1:**
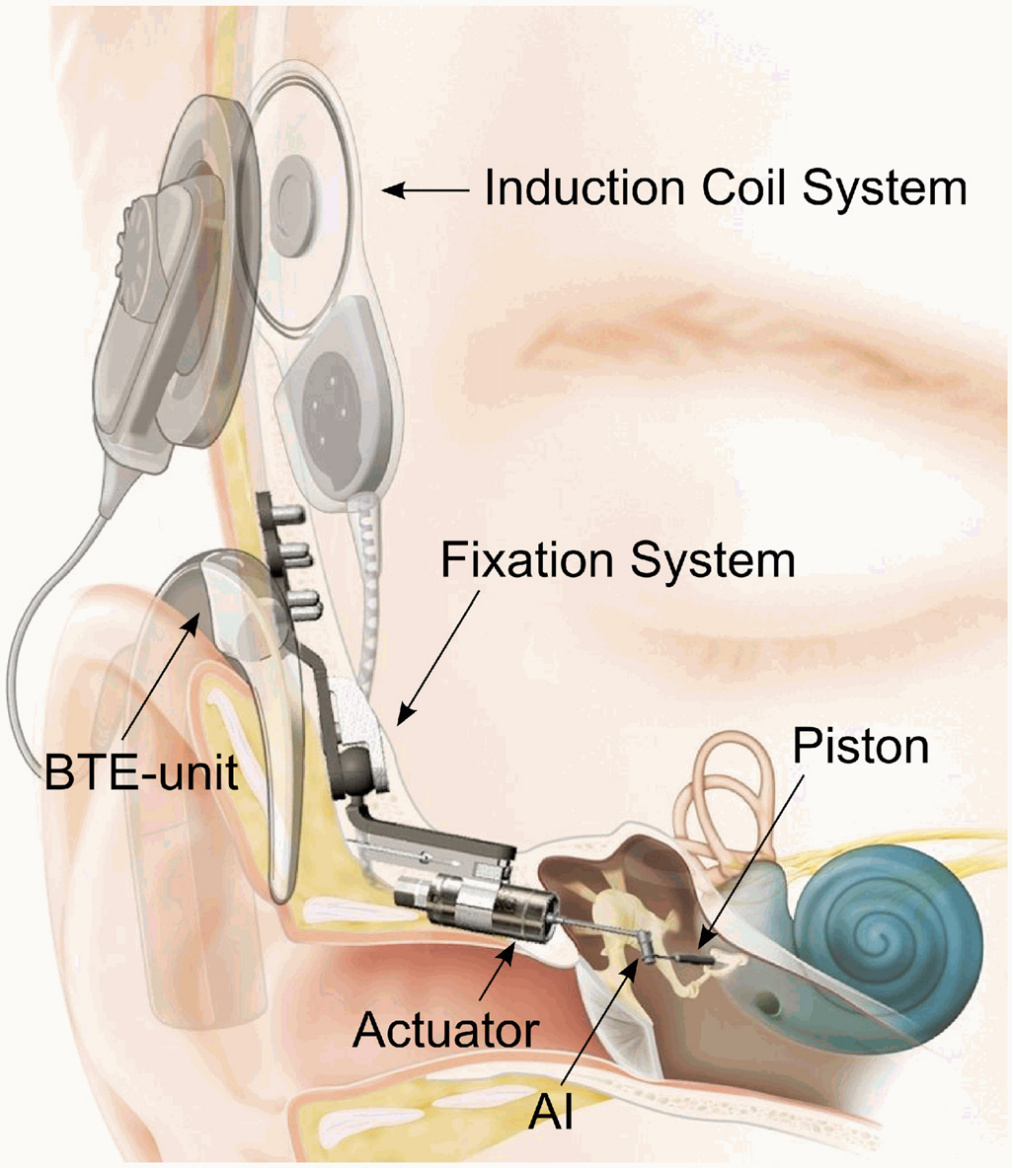
Illustration of the implanted Codacs system. (CC-BY by courtesy of Cochlear Ltd.).

The Codacs system (Cochlear Ltd., Sydney Australia) is a Direct Acoustic Cochlear Implant (DACI), stimulating the inner ear directly by vibration. The external behind-the-ear unit containing the sound processor receives the acoustical signal by two microphones (Fig. 1) and transmits it transcutaneously to the implant by an induction coil. The electro-magnetic Codacs actuator held by a fixation system (Fig. 1) generates the vibration. The vibration is transmitted to the perilymph fluid by a piston prosthesis crimped to the angled Codacs actuator rod tip, the artificial incus (AI), and inserted into the inner ear through a stapes footplate (SFP) fenestration. The direct stimulation of the perilymph bypasses the physiological sound transmission pathway and substitutes the middle ear and its ossicles.

The intended use of the Codacs System is the treatment of severe to profound mixed hearing losses caused by otosclerosis [1]. Its clinical applicability as well as that of a similar device have been demonstrated in earlier studies [2,3]. Beside the original use of the Codacs, further applications involving the stimulation of mobile middle ear structures or the round window (RW) are imaginable and would extend the indication range to patients with other pathologies. In particular, applications combining the advantages of a surgical reconstruction with an adjustable electro-mechanic amplification are advantageous in restoring the middle ear function [4]. Here the output of the Codacs actuator stimulating the stapes head (SH), the SFP and the RW was determined experimentally in human temporal bones (TBs) and compared to the perilymph piston stimulation.

### Codacs actuator

**Fig 2:**
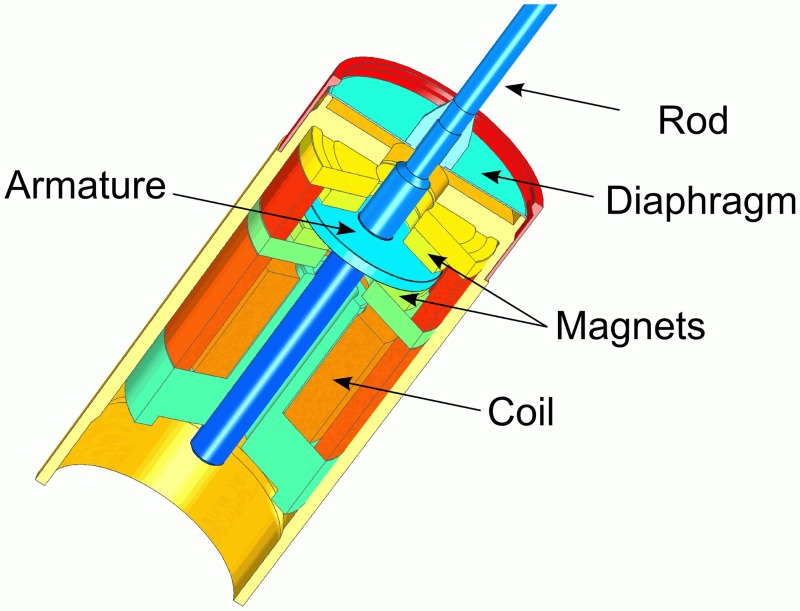
Section view of the Codacs actuator. (CC-BY by courtesy Cochlear Ltd.).

The Codacs actuator is an electromagnetic actuator based on the “balanced armature principle” [1]. As depicted in Fig. 2 a disk-like part of the rod inside the actuator is positioned between two permanent ring magnets. A titanium diaphragm encloses the rod and acts as a spring. The stiffness of the diaphragm is partially compensated by the force-displacement characteristics of the armature inside the magnetic field, resulting in a reduced dynamic stiffness of the ensemble and a corner frequency that is better adapted to that of the middle ear. An electromagnetic coil modifies the magnetic flux and thus induces axial vibrations of the rod. The mobile magnetic armature is exactly centered to result in a symmetric spring constant of the ensemble for perilymph piston stimulation when no static forces are applied to the vibrating rod [5].

Hence the Codacs actuator functioning crucially depends on the working point of the balanced armature. On the other hand stimulation of solid middle ear structures or the RW requires some static force preload for efficient transmission [6–9] that impacts the working point. Therefore the performance of the Codacs actuator may be impaired, when used in applications as in our experiments.

## Materials and Methods

### Determination of the static force working point

To estimate the output and distortion under preload with a defined static axial force, three bench tests were performed with Codacs actuators prior the TB experiments. A flexible plastic element was positioned in front of the actuator tip perpendicular to the actuator axis. By moving the actuator forward with a micromanipulator, axial static forces up to ~100 mN were applied stepwise in increments of 2.5 mN (1^st^ and 2^nd^ test) or 5 mN (3^rd^ test). The forces were measured by a force sensor (LSB210, FUTEK Advanced Sensor Technology, USA) mounted between actuator and micromanipulator. At every force level, the generated actuator displacement output was measured with a Laser Doppler Velocimeter (LDV) and the output amplitude was determined using a frozen pseudo random white noise input signal (25.6 kHz sample rate, 800 FFT lines) at approx. -50 dB re 1V_rms_/FFT line. To determine the total harmonic distortion (THD) a 0.6 and a 1 kHz sine signal of approx. -13 dB re 1V_rms_ electrical actuator input was used. From obtained displacement responses the THD was calculated using all available higher harmonics ≤ 10 kHz above noise level. In all three bench tests the actuator resonance frequency (RF) (unloaded ~ 2 kHz) increased with increasing force levels (212.5–437.5 Hz/50 mN) whereas the displacement amplitude at plateau range (< ~ 2 kHz) decreased only mildly (approx. -0.04 to -0.06 dB/mN). For all applied forces below the maximum of 100 mN the THD for 0.6 kHz input remained in a narrow range (1.7% to 2.1%), in contrast to THD for 1 kHz that amounted up to 7.4%. The higher THD of 1 kHz was attributed to the coincidence of the 1^st^ harmonic with the actuator RF at ~2 kHz, since it decreased when the RF was shifted upwards by higher loading (0.7–2.0% per 200 Hz RF-decrease). For the actuator stimulation experiments in TB we selected a static axial force load of 5 mN. This value was chosen for: (1) minimal decrease in output amplitude at low frequencies, (2) low RF shift (< 175 Hz), (3) tight physical contact, (4) sufficient sound transfer efficiency and (5) low force applied to the stimulated structures. The displacement outputs produced at ~ 5 mN by the Codacs driven by the 0.6 and a 1 kHz sine signals in bench tests are given in the supporting material (S1 Fig.—S6 Fig.).

### Temporal bone experiments

Experiments were performed in cadaveric human TBs obtained from the Institute for Pathology of the Hannover Medical School. Cadaverous tissues were used according to the legal rules of Lower Saxony after informed consent given by the relatives. Harvesting and anonymous use of TBs was approved by the local ethical committee of the Hannover Medical School (1963–2013). All preparations used for analysis fulfilled the modified acceptance criteria of “ASTM F2504–05 Standard Practice for Describing System Output of Implantable Middle Ear Hearing Devices (IMEHDs)” [10] by Rosowski et al. [11].

#### Preparation

All TBs were harvested within 48 h *post mortem* and stored at ~ -19°C before being thawed at room temperature shortly before preparation. An access to the RW and to the SFP was created by a mastoidectomy and dissection of the facial nerve. The pseudo membranes and the RW niche overhang except approx. 0.2–0.5 mm of the bony surrounding were removed for direct visual and mechanical access to the RW membrane. After preparation TBs were placed in saline containing ~ 0.005 ‰ thimerosal and stored at ~ 4°C until the experiment. In cases when the time between preparation and experiment exceeded ~ 15 h the TB in the saline solution was re-frozen at ~ -19°C and thawed immediately before experiments. During experiments the TBs were kept moist with saline to avoid changes in mechanical behavior [10].

#### Temporal bone response to sound measurements

The acceptance criterion for adequate TBs was SFP vibration measured in response to acoustical outer ear canal (OEC) stimulation. We selected the TBs using the extended acceptance range given by Rosowski et al. [11].

TBs were mounted in a laboratory clamp on a magnetic stand, placed on a vibration isolated table (LW3048B, Newport, Germany). An ear speculum was inserted into the OEC and sealed with dental cement (Paladur, Heraeus Kulzer GmbH, Germany). On this speculum a closed sound application setup with a probe microphone (ER-7C, Etymotic Research Inc., USA) was mounted. The probe microphone tip was placed 1–2 mm in front of the tympanic membrane and a loudspeaker (DT48, beyerdynamic, Germany) was connected to the sound application system with a tube.

For acoustical OEC stimulation the loudspeaker was driven by a buffer amplifier (SA1 Tucker-Davis Technologies, USA) with a custom written multi-sine signal, having equal amplitudes (approx. -25 dB re 1V_rms_) at 0.125, 0.25, 0.5, 1, 2, 3, 4, 6, 8 and 10 kHz. The signal was generated by a commercial 16 bit, 4-input channel data acquisition system (PC-D and VIB-E-400, Polytec, Germany) and a commercial data acquisition software (VibSoft 4.711, Polytec, Germany) using a 25.6 kHz sample rate. Input signals were acquired simultaneously as averaged complex spectra (800 FFT lines, 0–10 kHz, 12.5 Hz resolution, 500 avg.). The SFP and RW displacement responses were measured with a LDV system (CLV 700, controller HLV 1000, micromanipulator HLV MM2, Polytec, Germany) mounted on a microscope head (OPMI-1, Zeiss, Germany). To increase the laser beam reflection a small piece (< 0.3 mm x 0.3 mm) of reflective tape was placed on the SFP and RW. In contrast to the SFP, RW responses exhibit significant vibration modes at higher frequencies and vibration patterns may differ between preparations [12,13]. The RW vibration pattern was found variable with frequencies > 1.5 kHz [12,13], but independent of the acoustic stimulation level in the range between 80 and 110 dB SPL [13]. These results correspond to other findings that displacements measured at the center of the RW increases linear with acoustical stimulation level in the range 50–110 dB SPL [14]. Consequently vibration measurements at a single position cannot be used as indicator of absolute RW volume displacement, but the constancy of the vibration pattern allows a relative estimation of stimulation efficiency in forward stimulation. Hence, the reflector position on the RW was kept constant throughout each of our experiments at a position approx. halfway between the center and the edge. This specific position was chosen to allow the stimulation of the RW centrally without changing the position of the reflector. The visually estimated incident angles of the LDV laser beam were ≤ 60° to the SFP and ≤ 45°to the RW normal. During analysis a cosine correction to the measurement data was applied using the visually estimated angle. At each stimulation frequency the signal to noise ratio (SNR) was determined using the average of the three FFT lines below and above the specific frequency as noise level estimate. Responses with an SNR < 10 dB were excluded from analysis.

#### Codacs actuator stimulation

**Fig 3:**
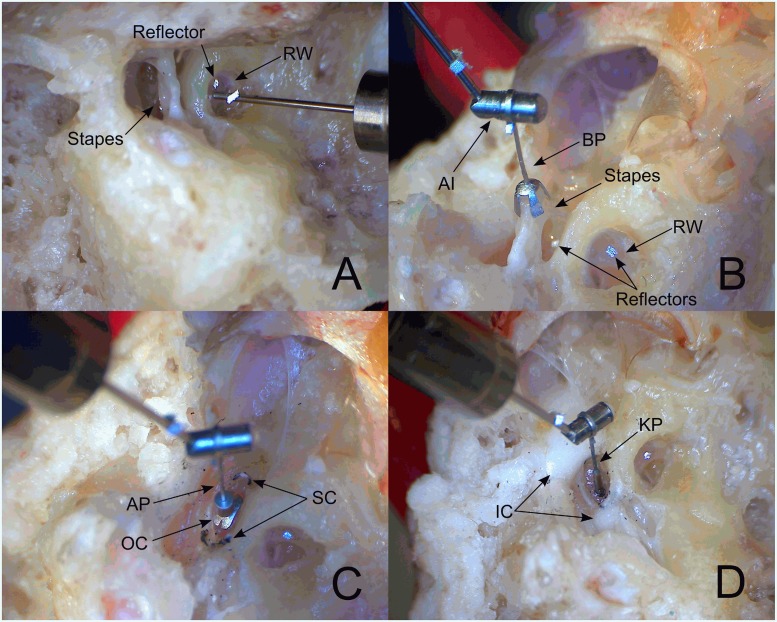
Stimulation modes tested. (A) Codacs actuator without AI perpendicular to the RW membrane. (B) Bell prosthesis (BP) crimped to the Codacs actuator AI and coupled to the exposed stapes head. Reflectors were placed on stapes footplate and round window (RW). (C) Omega connector (OC) placed between the remains of the stapes crura (SC) together with the Aerial prosthesis (AP) connected to the actuator AI. (D) K-piston (KP) inserted into the inner ear through a SFP fenestration after immobilization of the SFP by ionomer cement (IC).

In all experiments the Codacs actuator body was fixed to a rod mounted to a force sensor (LSB210, FUTEK Advanced Sensor Technology, USA) and the sensor was held by a three-axis micromanipulator (M3301R, World Precision Instruments Germany GmbH, Germany) on a magnetic stand. Hence, the actuator could be adjusted in all three spatial directions with the micromanipulator while controlling the axial force by the sensor. In experiments stimulating the OW or the perilymph directly, the Codacs with an artificial incus (AI) in combination with the respective prosthesis could be used. Experiments stimulating the RW were conducted with a modified actuator having no AI (see Fig. 3A). The actuator was electrically driven with the same multi-sine signal at approx. -30 dB re 1V_rms_, previously used for the acoustical stimulation with the loudspeaker.

For RW stimulation experiments (N = 10) the tip of the actuator rod (diameter 0.4 mm), having no sharp edges, was directed to the center of the RW with the micromanipulator perpendicular, and the axial coupling force was adjusted to ~ 5 mN (Fig. 3A). When the RW stimulation experiment was finished, a direct access to the stapes was created for the following stimulation experiments by an incudectomy and removal of the malleus, the outer ear canal and the tympanic membrane.

In experiments stimulating the SH (N = 9), a titanium Bell prosthesis (length: 3.0 mm, Heinz Kurz GmbH, Germany) was manually crimped to the AI. The bell-shaped end of the prosthesis was plugged onto the exposed SH (Fig. 3B). As before, the axial preload was adjusted to ~ 5 mN and lateral forces were minimized by avoiding visible tilting of the stapes.

In experiments stimulating the SFP (N = 8) the stapes suprastructure was removed with a surgical diode laser (Iridis, Quantel Medical, France) and a titanium Omega connector (Heinz Kurz GmbH, Germany) was placed on the SFP between the remains of the crura (Fig. 3C). The loop of a titanium Aerial prosthesis (length: 5.5 mm, Heinz Kurz GmbH Medizintechnik, Germany) was manually crimped to the actuator AI and the cylindrical prosthesis end was plugged onto the ball of the omega connector to form a ball joint. The adjustment of the axial coupling force to ~ 5 mN and lateral forces minimization were done as before.

In direct perilymph stimulation experiments (N = 10), otosclerosis was simulated by immobilizing the SFP with ionomer cement (Denseal Superior, Prevest Denpro GmbH, Germany) and a stapedotomy was performed with the surgical laser. In a pilot experiment we checked that the vibration of the promontory and the fixated SFP was at least-60 dB attenuated compared to the vibrating piston and actuator (data not shown). A titanium K-Piston prosthesis (0.4 x 5.0 mm, Heinz Kurz GmbH Medizintechnik, Germany) was manually crimped to the Codacs AI before it was inserted into the cochlear through the fenestration of the SFP (diameter ~ 0.5–0.6 mm) (Fig. 3D). In this condition the axial coupling force was ~ 0 mN, while lateral forces were minimized by centering the piston in the opening perpendicular to the SFP. In all TBs the sequence of performed stimulation modes was (1) RW stimulation, (2) Bell prosthesis, (3) Omega/Aerial prosthesis and (4) finally K-piston, except in experiment TB12 where the Omega/Aerial prosthesis mode was omitted.

According to the ASTM standard F2504–05 [10], stapes vibration responses to sound and actuator stimulation are used as reference for IMEHD output determination. In the K-piston stimulation mode the SFP was immobile and not directly actuated, and when stimulated with the Omega/Aerial prosthesis the SFP was mostly occluded. Only in Bell prosthesis stimulation and RW stimulation the SFP was visually accessible and could be used to determine the actuator output. Due to this restriction we used the RW vibration measured at a fixed position in response to sound and actuator stimulation as alternative reference for actuator output determination where no SFP response was usable. When both the SFP and the RW could be used (Bell prosthesis) both sites were measured to test the equivalence. In case of the Omega prosthesis and the K-piston, vibration in response to sound and actuator stimulation on the identical site on the RW membrane was used as references throughout the experiment. In the analysis all displacement outputs were normalized for 1 V_rms_ actuator input voltage assuming a linear dependency from the input voltage.

To verify that actuators were within the specifications of the manufacturer, the unloaded resonance frequency was determined before each stimulation mode and compared to the acceptance range (RF ≤ 2.5 kHz; difference RF-RF_manufacturer baseline_ ≤ 300 Hz, Cochlear Ltd.). For this pre-experimental testing the unloaded actuator was driven with the same white noise input used in the bench tests and the displacement output was measured at the rod. The total duration of TB experiments was between ~ 3.5 h and ~ 6.5 h.

#### Equivalent sound pressure level determination

The ASTM standard F2504–05 [10] specifies a method to convert measured stapes vibration responses of IMEHDs to equivalent sound pressure levels (eq. dB SPLs). As described above, the visual access to the SFP, needed for this purpose, was not given in all experimental conditions. In these cases the RW response was measured instead and used to calculate eq. dB SPL. In case of stimulation with the Bell prosthesis both reference measurements were possible and both were performed for comparison. In RW stimulation experiments the SFP displacement was used as indicator of relative output.

Before implantation the displacement of the stapes ($d_{U}^{SFP}$) or RW ($d_{U}^{RW}$) in response to acoustical stimulation at the tympanic membrane (sound pressure *p*_*T*_) were recorded and the middle-ear transfer functions $H_{TV}^{SFP}$ (1a) and $H_{TV}^{RW}$ (1b) were determined:

$$H_{TV}^{SFP}=d_{U}^{SFP}/p_{T}H_{TV}^{RW}=d_{U}^{RW}/p_{T}$$ (1a, b)

Similarly, the electro-vibrational transfer functions $H_{EV}^{SFP}$ (2a) and $H_{EV}^{RW}$ (2b) were determined as the ratio of the displacements of the stapes ($d_{A}^{SFP}$) and the RW ($d_{A}^{RW}$) and the electrical input *E to* the actuator:

$$H_{EV}^{SFP}=d_{A}^{SFP}/EH_{EV}^{RW}=d_{A}^{RW}/E$$ (2a, b)

Having *H*_*TV*_ and *H*_*EV*_, the equivalent ear canal sound pressure transfer function can be computed using either the SFP vibration $H_{EV}^{SFP}$ (3a) or the RW vibration $H_{EV}^{RW}$ (3b) as reference:

$$H_{ET}^{SFP}=H_{EV}^{SFP}/H_{TV}^{SFP}H_{ET}^{RW}=H_{EV}^{RW}/H_{TV}^{RW}$$ (3a, b)

With the maximum electrical actuator input (*E*_max_), the maximum equivalent sound pressure level (*L*_max_) can be determined in two different ways using either the SFP (4a) or the RW (4b) vibration as reference:

$$L_{E_{\max}}^{SFP}=20\log_{10}\left(H_{ET}^{SFP}\times E_{\max}/2\times 10^{-5}\text{Pa}\right).$$ (4a)

$$L_{E_{\max}}^{RW}=20\log_{10}\left(H_{ET}^{RW}\times E_{\max}/2\times 10^{-5}\text{Pa}\right).$$ (4b)

As mentioned before all responses have to be measured at the identical site on the RW since the validity of results depends on the constancy of the vibration pattern in all stimulation modes due to the relative nature of the vibration output at the RW.

For the analysis a hypothetical electrical input to the actuator of *E*_max_ = 1 V_rms_ was assumed to determine the maximum eq. sound pressure level output.

## Results

### Temporal bone responses to sound

**Fig 4:**
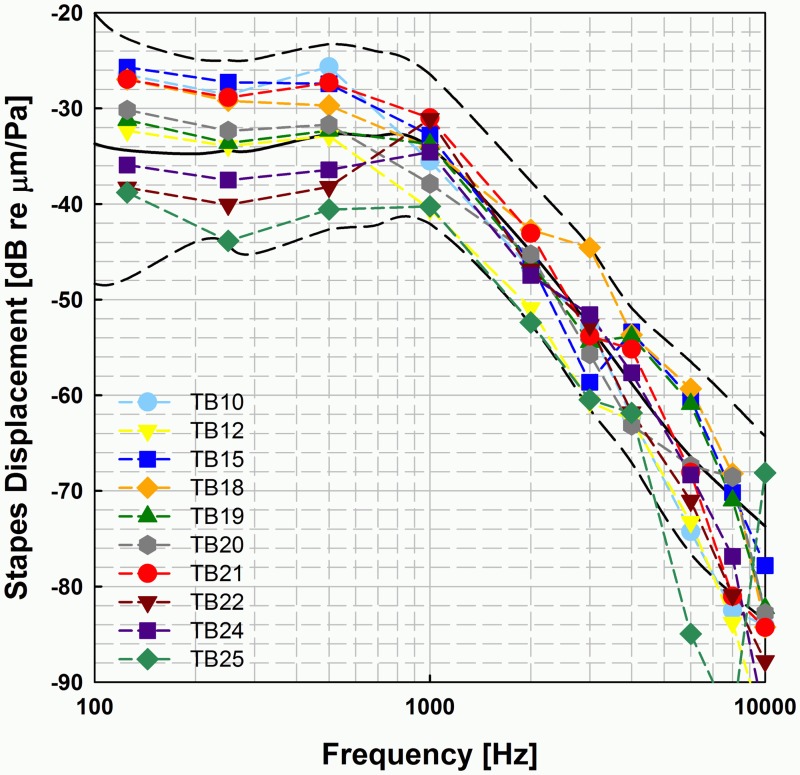
SFP displacement responses to sound of TBs used for experiments (N = 10). The black dashed lines depict the limits given by Rosowski et al. [11].

Fig. 4 summarizes the SFP displacement responses to sound [dB re μm/Pa] of the TBs used in the analysis of Codacs actuator stimulation experiments. In the frequency range between 0.25 to 4 kHz 10 out of 25 preparations were found within the acceptance range given by Rosowski et al. [11] and contributed data to the following analysis. The RW displacement responses to sound are likewise depicted in the supporting material S7 Fig.

### Codacs stimulation outputs

As described above, all Codacs outputs were calculated and compared in this work in terms of eq. SPL. Raw displacements data measured at the SFP and at the RW in response to the actuator stimulation can be found in the supporting material S8 Fig. to S12 Fig.

#### Oval window stimulation

**Fig 5:**
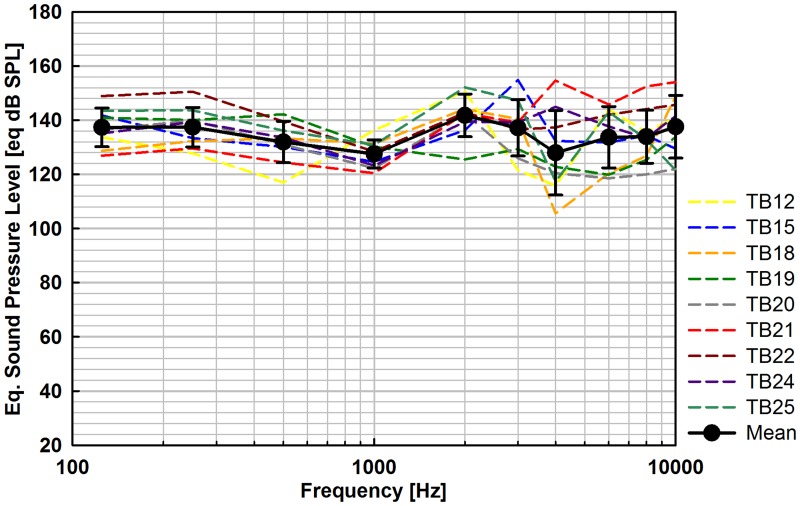
Eq. sound pressure output level (N = 9) of Codacs stimulation with the Bell prosthesis at the SH for nominally 1 V_*rms*_ actuator input voltage.

**Fig 6:**
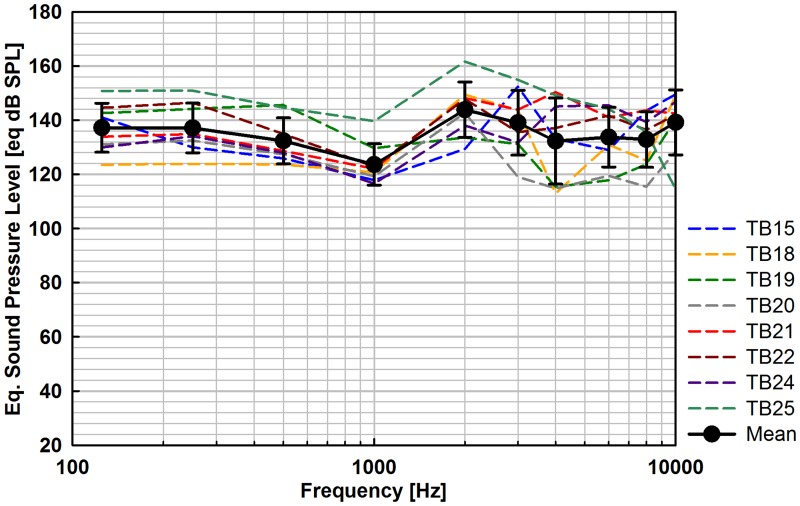
Eq. sound pressure level output (N = 8) of Codacs stimulation with the Omega/Aerial prosthesis at the SFP for nominally 1 V_*rms*_ actuator input voltage.

The eq. SPL results, stimulating with the Bell prosthesis (Fig. 5) and the Omega/Aerial prosthesis (Fig. 6) were of similar flat shape and increasing spread at frequencies > 1 kHz. For Bell stimulation, mean outputs were between 127.5 and 141.8 eq. dB SPL and for Omega/Aerial stimulation between 123.6 and 143.9 eq. dB SPL. The average output level at speech relevant frequencies (avg. 0.5, 1, 2, 3, 4 kHz) was 133.3 eq. dB SPL with the Bell and 134.2 eq. dB SPL with the Omega/Aerial prosthesis.

#### RW stimulation

**Fig 7:**
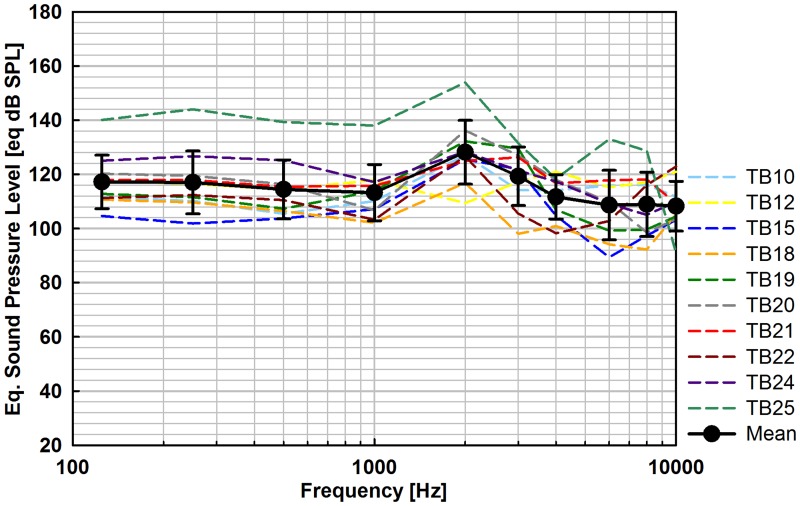
Eq. sound pressure level output (N = 10) obtained in Codacs stimulation of the RW membrane at nominally 1 V_*rms*_ actuator input voltage.

Across all experiments stimulating the RW eq. SPL outputs were of similar shape and in the range between 89.4 and 136.3 eq. dB SPL (Fig. 7). In experiment TB25 the output at frequencies ≤ 2 kHz was distinctly higher (138.0–154.0 eq. dB SPL). The mean eq. SPLs were 108.3–128.2 eq. dB SPL and the average output level at speech relevant frequencies (avg. 0.5, 1, 2, 3, 4 kHz) was 117.4 eq. dB SPL at 1V_rms_ input.

#### K-piston stimulation

**Fig 8:**
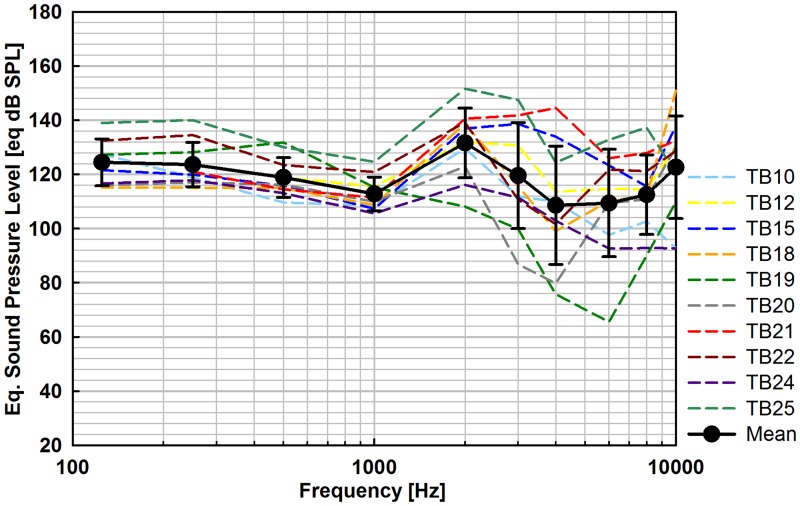
Eq. sound pressure level outputs (N = 10) of Codacs stimulation through a SFP fenestration with a K-piston at nominally 1 V_*rms*_ actuator input. Data having SNRs < 10 dB was omitted (TB12 and TB21 at 125 Hz).

The Eq. SPL output produced by the Codacs actuator when stimulating the inner ear directly with a K-piston were flat at frequencies ≤ 1 kHz showing an increased spread at higher frequencies (Fig. 8). Except in two experiments (TB19, TB20) all obtained SPL were > 90 eq. dB SPL in the entire frequency range. At frequencies ≤ 1 kHz the mean eq. SPL was between 112.8 and 124.5 eq. dB SPL and at frequencies > 1 kHz between 108.6 and 131.6 eq. dB SPL with standard deviations up to 21.9 dB. The average output at speech relevant frequencies (avg. 0.5, 1, 2, 3, 4 kHz) was 118.3 eq. dB SPL. Measurements at 125 Hz in experiments TB12 and TB21 with a SNR < 10 dB were omitted.

#### Comparison of stimulation outputs

**Fig 9:**
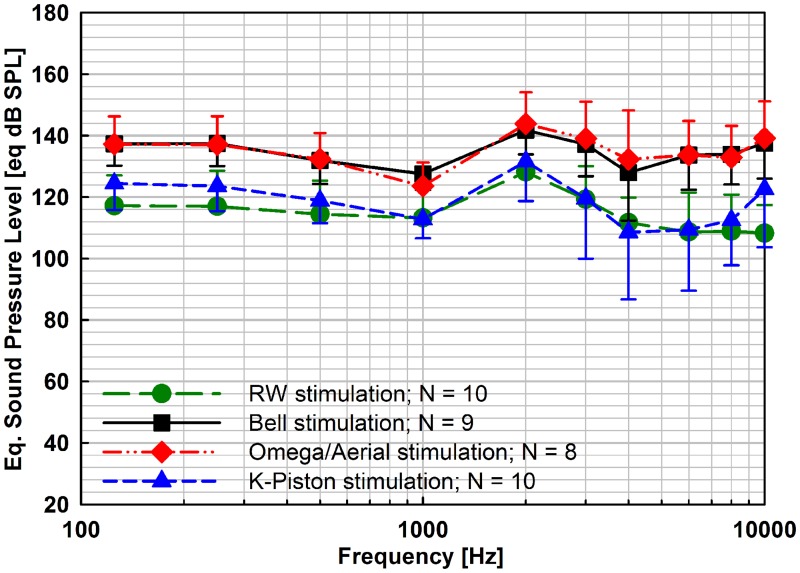
Mean values and standard deviations (error bars) of the equivalent sound pressure levels [eq. dB SPL] generated by Codacs actuator stimulation at 1 V_rms_ for the four investigated stimulation modes. At all frequencies the stimulation efficiency of Bell prosthesis stimulation at the stapes head (■) and of Omega/Aerial prosthesis stimulation at the SFP (◆) was statistically significantly higher than direct perilymph stimulation (▲) with the K-piston and direct RW stimulation (●).

In all stimulation modes the average eq. SPL outputs were of similar shape with a minimum-maximum difference < 23.0 dB (Fig. 9, Table 1). A peak response occurred in all stimulation modes at 2 kHz which corresponds to the approximate actuator resonance frequency. Both, Bell stimulation and Omega/Aerial stimulation provided similar outputs with statistically not significant mean differences of maximal 3.2 dB (two-tailed paired t-tests, Table 2). Compared to K-piston stimulation, the output involving the entire SFP was statistically significant higher (Bell: 10.0 to 23.0 dB; Omega/Aerial: 10.6 to 24.5 dB) (Kolmogorov-Smirnov test; one-tailed paired t-test, Table 2) at most frequencies. Only exceptions were the difference Bell vs. K-Piston at 6 kHz and Bell vs. Aerial/Omega at 4 kHz (Wilcoxon Signed Rank Test).

**Table 1:** Mean equivalent output levels and standard deviations of all stimulation modes tested.

	Bell		Omega/Aerial		RW		K-Piston	
Frequency	Mean	SD	Mean	SD	Mean	SD	Mean	SD
[kHz]	[eq dB SPL]	[dB]	[eq dB SPL]	[dB]	[eq dB SPL]	[dB]	[eq dB SPL]	[dB]
**0.125**	137.344	7.12	137.20	9.03	117.22	9.90	124.46	8.64
**0.25**	137.418	7.28	137.14	9.17	117.04	11.60	123.64	8.18
**0.5**	131.934	7.65	132.36	8.53	114.45	10.86	118.86	7.33
**1**	127.543	5.19	123.62	7.61	113.24	10.38	112.80	6.21
**2**	141.79	7.86	143.87	10.23	128.16	11.76	131.61	12.86
**3**	137.173	10.40	139.06	11.95	119.28	10.78	119.54	19.55
**4**	127.963	15.61	132.28	15.89	111.68	8.17	108.57	21.86
**6**	133.7	11.32	133.73	11.01	108.67	12.83	109.42	19.85
**8**	133.913	9.79	132.87	10.28	108.93	11.87	112.49	14.67
**10**	137.57	11.56	139.18	12.01	108.25	9.21	122.64	18.95

**Table 2:** Statistical analysis of differences in eq. output level [eq. dB SPL].

Frequency	Bell vs. K-Piston			Omega/Aerial vs. K-Piston			Bell vs. Omega/Aerial			RW vs. K-Piston			RW vs. Omega/Aerial			RW vs. Bell		
	Mean Difference	p-Value	N	Mean Difference	p-Value	N	Mean Difference	p-Value	N	Mean Difference	p-Value	N	Mean Difference	p-Value	N	Mean Difference	p-Value	N
[kHz]	[dB]	[-]	[-]	[dB]	[-]	[-]	[dB]	[-]	[-]	[dB]	[-]	[-]	[dB]	[-]	[-]	[dB]	[-]	[-]
**0.125**	15.34	0.000168	7	13.65	0.000021500	7	0.61	0.761	8	-7.39	0.103	8	-19.34	0.0012900	8	-19.51	0.00069100	9
**0.25**	13.26	0.000216	9	12.91	0.000002140	8	1.48	0.515	8	-6.60	0.070	10	-19.17	0.0008250	8	-19.62	0.00059300	9
**0.5**	12.05	0.000379	9	12.35	0.000000671	8	1.44	0.528	8	-4.94	0.231	10	-16.79	0.0021800	8	-16.49	0.00288000	9
**1**	14.26	0.000042	9	10.64	0.000068600	8	2.82	0.229	8	0.43	0.880	10	-10.59	0.0027000	8	-13.97	0.00274000	9
**2**	9.96	0.006900	9	12.07	0.006760000	8	-3.20	0.143	8	-3.45	0.513	10	-13.24	0.0057300	8	-13.52	0.01180000	9
**3**	16.79	0.008570	9	19.98	0.000753000	8	0.05	0.978	8	-0.26	0.970	10	-18.89	0.0086500	8	-17.32	0.00484000	9
**4**	19.54	0.012900	9	24.52	0.001930000	8	-2.81	1.00^(1^	8	3.11	0.663	10	-22.07	0.0018200	8	-16.55	0.00975000	9
**6**	22.98	0.001710	9	23.50	0.00800000^(1^	8	-1.31	0.505	8	-0.74	0.909	10	-26.79	0.0002710	8	-25.83	0.00006910	9
**8**	20.33	0.000934	9	19.42	0.005140000	8	0.83	0.724	8	-3.56	0.385	10	-25.88	0.0002390	8	-25.79	0.00003100	9
**10**	11.61	0.048600	9	13.77	0.045800000	8	-2.04	0.577	8	-14.40	0.063	10	-32.96	0.0000145	8	-29.71	0.00000609	9

A paired t-test was used except in rare cases where the differences were not normally distributed (Kolmogorov-Smirnov test) and a non-parametric test (Wilcoxon Signed Rank Test) was used^1^.

RW stimulation provided similar outputs to K-piston stimulation with small (≤ 7.4 dB excepting 14.4 dB at 10 kHz), statistically not significant differences (Kolmogorov-Smirnov test; two-tailed paired t-test, Table 2). At speech relevant frequencies (0.5–4 kHz) the average difference was ≤ 4.9 dB. In both modes, stimulating the entire SFP (Bell and Omega/Aerial) the output was 10.6 to 33.0 dB higher than in RW stimulation, being significant at all frequencies (Kolmogorov-Smirnov test; one-tailed paired t-test, Table 2).

#### Comparison of RW and SFP as output reference

**Fig 10:**
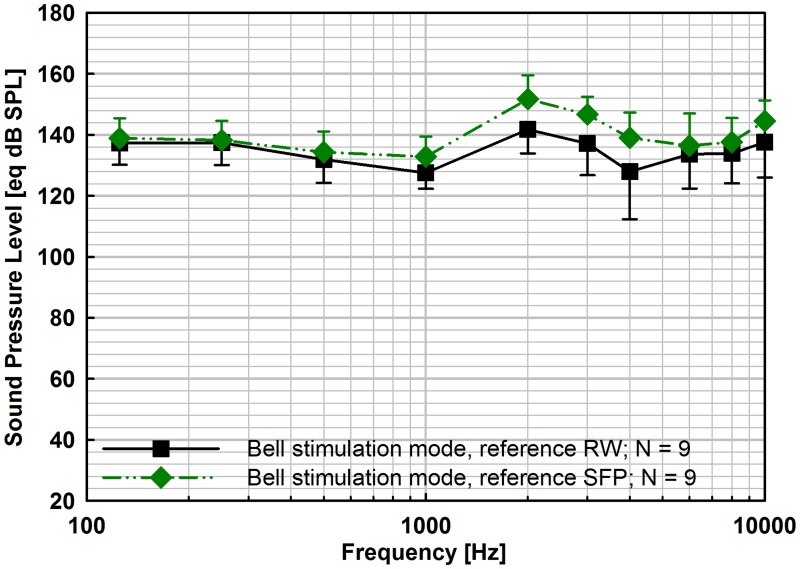
Equivalent sound pressure levels obtained by oval window stimulation with the Bell prosthesis using either the RW displacements (■) or the SFP displacements (◆) in response to sound and actuator as reference. Mean values and standard deviations (error bars) of eq. sound pressure level are shown for nominally 1 V_rms_ actuator input.

Mean eq. SPL outputs in Bell stimulation mode calculated from the displacements of the SFP and the RW are in good accordance over the entire frequency range with slightly higher results using the SFP as reference (Fig. 10). The difference between the eq. dB SPL calculated from vibrations at both anatomical locations was significant at 1, 2, 3 kHz (Kolmogorov-Smirnov, Paired t-test) with mean differences < 10 dB and at maximum of 11.1 dB (4 kHz, p = 0.156). At low (< 1 kHz) and high frequencies (> 4 kHz) differences were even smaller (< 6.9 dB).

## Discussion

In human temporal bone preparations we performed Codacs actuator stimulations under constant axial force with (1) a Bell prosthesis at the SH, (2) an Omega connector at the SFP in combination with an Aerial prosthesis and (3) directly at the RW. The actuator output expressed as eq. sound pressure level was estimated using RW displacement and/or SFP displacement responses and was compared to direct perilymph stimulation with a K-piston.

### Codacs usability with static force preload

In bench experiments the Codacs actuator displacement output in the plateau range (~ < 2 kHz) showed only a mild decrease with increasing static force applied to the actuator up to the maximum of approx. 100 mN. Also the THD remained at levels below 2.1% when tested with 600Hz. Higher THDs using a stimulus frequency of 1kHz were attributed to the coincidence of the 1^st^ harmonic with the resonance frequency of the actuator and decreased with the applied force. In earlier performed RW stimulation experiments with an actuator of similar design (DACS PI, Phonak Acoustic Implants SA, Switzerland) the impact of static preload on the output amplitude and RF was found minor at forces < 37.2 mN [6]. These findings demonstrate that the Codacs actuator originally designed for stimulations without static force load may also be suitable for applications requiring some axial forces to the actuator rod. The used static preload in our experiments was chosen not only to achieve minimum output reduction, THD and maximum coupling efficiency, but also to remain with the SFP vibration in a linear range. Because the SFP has a linear force-displacement behavior up to approx. 10–15 mN of static load [15], a static preload of 5 mN was taken. Although the Codacs was specifically designed to be coupled to the perilymph [5] where no static force preload is expected, our bench experiments indicate that it can be used in applications applying a static force preload with sufficiently high output amplitude and low distortion (see suppl. material S1—S6 Figs.). Whereas the actuator can be used over a wide range of static forces, limitations are more likely due to force limits by the stimulated structure, for instance saturation of the SFP.

### RW and SFP single point LDV measurements as reference for output determination

To assess the error resulting from single point measurements of RW vibration responses as reference, we compared the mean eq. SPLs of the Bell stimulations calculated from RW and SFP vibrations. Similar to Devèze et al. [16] the eq. SPL was higher when using SFP vibrations as reference in Bell stimulation with a T2 MET actuator (2^nd^ generation Middle Ear Transducer, Otologics Boulder, USA), although the difference was more pronounced (~ 2–23 dB) than in our experiments (0.9–11.1 dB). Stenfelt et al. [12] determined a difference in volume displacement between OW and RW in response to acoustical stimulation of ≤ 3 dB using a 5 point measurement matrix on the SFP and a 27–40 point matrix with 0.2 mm spacing on the RW to determine the integral volume displacement. In experiments stimulating the SFP directly a similar difference (~ 3 dB) in volume displacement between OW and RW was found between 0.06 and 1.5 kHz using an acoustic probe at the RW [17]. Equally small differences (< 3 dB) we determined between eq. SPLs calculated by SFP and RW vibrations at 0.125–0.5 kHz, and 6 kHz. A possible origin of bigger differences (3.8–11.1 dB) found in our results at the other frequencies (1–4 kHz, 8–10 kHz) may be due to rocking motions of the stapes undetected by our single point measurement at sites close to the anterior crus. In case of the actuator stimulation the Bell prosthesis was aligned perpendicular to the SFP (see Fig. 3) and to the AI. The angle of 25° between the AI to the actuator axis may have led to tilting of the SFP and to an overestimation of the volume displacement at the out-of-center measurement target in the direction of the long SFP axis. However the limitation in eq. sound pressure output level difference determined by RW and SFP responses indicate that both sites can be used as reference to estimate the output level, provided that the measurement target is identical for acoustical stimulation and actuator stimulation.

### OW stimulation with Codacs

When the SH or the SFP is stimulated, the entire OW acts as the mechanical input to the inner ear. Therefore it can be assumed that both stimulation modes produce equally outputs. Being statistically indistinguishable, the here determined eq. SPLs of Codacs stimulation with the Bell and Omega/Aerial prostheses confirm this presumption. The obtained output of the Codacs actuator in Bell stimulation mode was higher than the output generated by a T2 MET actuator (2^nd^ generation Middle Ear Transducer, Otologics Boulder, USA) in a similar stimulation experiment [16]. Between 0.25 and 8 kHz the MET provided an output of ~ 113 to ~ 138 eq. dB SPL compared to the Codacs that provided 127.5 to 141.8 eq. dB SPL, at 1 V_rms_ input voltage. Since the prosthesis was bent and coupled to the SH in another angle in experiments with the T2 MET and no information about the static coupling force is provided, the results of both actuators are not strictly comparable.

### RW stimulation

In RW stimulation the output of the device was exclusively determined using SFP vibration as reference. Although this method is commonly used for output determination of actuators stimulating the RW [6,18,19], it is subject to restrictions. Due to the higher total acoustic impedance in reverse RW stimulation than in forward OW stimulation the stapes vibration amplitude is expected to be smaller in RW stimulation compared to OW stimulation. Investigations by Stieger et al. [20] measuring the stapes velocity and intracochlear pressures during forward stimulation and reverse stimulation of the RW (0.1 to 10 kHz) confirmed this assumption. Normalized to the same inner ear input (pressure difference between both scalae) the stapes velocity was less in RW stimulation than in acoustical stimulation, most pronounced at frequencies < 1 kHz. However, an electro-physiological study in guinea pigs measuring auditory brainstem responses (ABRs) [21] in acoustic and RW stimulation points in the opposite direction. At a similar SFP velocity lower ABR amplitudes and longer latencies indicate a lower efficiency in reverse (RW) stimulation and consequently an overestimation of the physiological input in experiments using SFP vibration amplitude as reference. Beside differences in mechanical properties of guinea pig and human RW membranes the unknown pre-stress in earlier experiments may account for the disagreement, emphasizing the importance of well-defined coupling conditions. Here calculated eq. SPLs shall serve as approximation of the obtained output in reverse stimulation of the cochlea.

Comparison with the eq. sound pressure output level generated by the DACS PI [6] and MET actuator [19] estimated from stapes vibrations, shows that the Codacs actuator is similar or more efficient in RW stimulation. In contrast to the DACS PI (spherical prosthesis, ⌀ 0.5 mm, ~4 mN) the Codacs output level shows no roll-off at frequencies > 2 kHz (Fig. 7) in RW stimulation. The output of both actuators were similar at frequencies ≤ 1 kHz (Codacs: 113–117 eq. dB SPL, DACS PI: 110–115 eq. dB SPL), whereas the Codacs output (108–119 eq. dB SPL) was substantially higher than the DACS PI output (90–105 eq. dB SPL) at higher frequencies (> 2 kHz). Compared to RW stimulation with the MET T1 (0.5 mm spherical tip, estimated “several hundred dynes” force load (100 dyne = 1 mN)), averaged output at low- (0.25–1 kHz), mid- (1–3 kHz) and high-frequencies (3–8 kHz) of the Codacs (low: 115, mid: 120 and high: 112 eq. dB SPL) was also substantially higher than of the MET (low: 95, mid: 95 and high 109 eq. dB SPL) [19].

### Piston stimulation with Codacs

Estimating the eq. SPL of direct perilymph stimulation from single point RW vibration is subjected to limitations due the SFP fenestration. As described before the validity of using the RW response at a single site requires that the RW vibration pattern remains unchanged and that it is independent from stimulation level. According to Stenfelt et al. [13] this can be assumed for an intact SFP but fails in case of a stapedotomy and piston insertion. After a stapedotomy and piston prosthesis insertion Stenfelt et al. found at frequencies < 1.5 kHz a moderate change in RW vibration pattern (approx. -3 to -14 dB at 0.1–0.5 kHz and of approx. +/- 5 dB at 0.5–1.5 kHz) after piston insertion. At higher frequencies the amplitude and phase of the targets changed strongly (~ 10 dB to ~ -15 dB) without a trend across targets. Corresponding to these findings the eq. SPLs obtained here in the K-piston stimulation showed little variability at frequencies ≤ 1 kHz but an increased spread at higher frequencies (Fig. 8, Table 1). Hence the here determined eq. SPL of K-piston stimulation can be used for comparison at frequencies ≤ 1 kHz. At higher frequencies the results may serve as an estimate.

The output generated by the DACS PI with piston stimulation was determined by Chatzimichalis et al. [22] reconstructing the integral RW volume displacement from multi-point LDV measurements. They determined an output level of approx. 118–126 eq. dB SPL at 0.3 V_rms_ input in the range between 0.125 and 1 kHz. Considering a ~4 dB difference, expected theoretically from the different piston diameters used (DACS PI: ⌀ 0.5 mm; Codacs: ⌀ 0.4 mm), the DACS PI is approx. 8–11 dB more efficient. As in RW stimulation the Codacs shows no roll-off at frequencies ≥ 2 kHz that is seen in the DACS PI [6], leading to an approx. 14 dB higher output at 10 kHz. In a similar piston stimulation experiment [16] the averaged output of a T2 MET at 1 V_rms_ input was 106, 119 and 101 eq. dB SPL at low- (0.25–1 kHz), mid- (1–3 kHz) and high-frequencies (3–8 kHz). Considering a ~4 dB difference, expected theoretically from the different piston diameters used (T2 MET: ⌀ 0.5 mm; Codacs: ⌀ 0.4 mm), the Codacs is approx. 16, 2, 12 dB (low-, mid-, high-frequencies) more efficient.

### Comparison of stapes, RW and piston stimulation efficiencies with Codacs

The Codacs actuator stimulation of the SFP with the Bell prosthesis or Omega/Aerial prosthesis was statistically significant more efficient than the K-piston stimulation through a SFP fenestration. At frequencies ≤ 1 kHz both methods provided 10.6–15.3 dB higher outputs (Table 2). At higher frequencies, where the determined K-piston output serves as estimation, the output in OW stapes stimulation was 10.0–24.5 dB higher. These differences are less than the 28 dB difference of volume displacement that are theoretically expected from the ratio of the K-piston area (0.1257 mm^2^, ⌀ 0.4 mm) to the SFP area (3.2 mm^2^ [23]). This suggests that the output was probably affected by further aspects beside the volume displacement difference resulting from the area inducing mechanical stimulation. On the other hand, a comparison with the theoretical difference is generally only possible to a limited extent since the eq. SPL calculated for the performed K-piston stimulation may be effected by the disturbance of the SFP integrity.

The RW stimulation output was similar to the K-piston stimulation output with no statistically significant differences between each other. Compared to the Bell and Omega/Aerial stimulation mode, the RW stimulation was statistically significantly less efficient (10.6–33.0 dB). However it has to be considered that the obtained RW stimulation eq. SPL output probably underestimate the real Codacs actuator output. Even if their outputs differ in efficiency, all four tested stimulation modes are, based on our results, usable with the Codacs actuator since all eq. dB SPL were sufficient for hearing aid applications. Due to the mentioned limitations of the single-point LDV measurements, multi-point LDV measurements or intracochlear pressure measurements are necessary to determine output level, comparable in all stimulation modes over the entire frequency range.

### Feasibility of alternative stimulation modes

Although our results show that alternative stimulation sites can be successfully addressed with the Codacs actuator it must be emphasized that the experimental conditions were optimized and differ essentially from the clinical situation *in vivo*. Our specific experimental configuration was selected to control variables (e.g. static force and geometrical constrictions) that potentially influence the stimulation. For clinical applications this approach has to be adapted to the anatomical constraints. The angle between the actuator axis and the normal of the SFP used in the Bell, Omega/Aerial and K-piston stimulation modes was comparable to *in vivo* applications. In contrast, a coupling of the actuator rod perpendicular to the RW membrane is not feasible *in vivo*. Due to anatomical constraints a shallower angle has to be expected in clinical RW applications which might cause lateral forces to the actuator rod and a decrease in the efficiency of the RW stimulation mode. When a Bell or Aerial prosthesis is crimped to the AI of the Codacs the total length exceeds potentially the space available *in vivo*. Therefore a redesign of the Codacs rod might be necessary for these applications. To achieve *in vivo* force-controlled coupling conditions similar to our experiments, a mechanism to determine at least the axial component of the loading force has also to be implemented.

## Conclusion

The performed experiments demonstrated that the Codacs actuator, originally designed for applications without static axial forces to the rod, is usable under an axial preload of ~ 5 mN. All investigated stimulation modes provided sufficient output for hearing aid applications when the stimulation was performed at a controlled preload of ~ 5 mN (Bell, Omega/Aerial and RW mode) with output levels equal or higher than in K-piston stimulation without static force preload. Stimulation of the SFP using a Bell or Omega/Aerial prosthesis increased the efficiency compared to RW or direct stimulation with a K-piston. Furthermore the results demonstrate that single-point displacements responses of RW and SFP are both adequate references to determine the eq. SPL output of actuator stimulations at the intact stapes, providing that the measurement target is kept constant.

## Supporting Information

S1 Fig Displacement output of the Codacs actuator driven by an electrical 0.6 kHz sine wave input signal in the 1^st^ bench test at a static preload of ~ 5mN. THD = 1.7%
(PDF)

S2 Fig Displacement output of the Codacs actuator driven by an electrical 1 kHz sine wave input signal in the 1^st^ bench test at a static preload of ~ 5mN. THD = 6.5%.
(PDF)

S3 Fig Displacement output of the Codacs actuator driven by an electrical 0.6 kHz sine wave input signal in the 2^nd^ bench test at a static preload of ~ 5mN. THD = 2.1%.
(PDF)

S4 Fig Displacement output of the Codacs actuator driven by an electrical 1 kHz sine wave input signal in the 2^nd^ bench test at a static preload of ~ 5mN. THD = 5.2%.
(PDF)

S5 Fig Displacement output of the Codacs actuator driven by an electrical 0.6 kHz sine wave input signal in the 3^rd^ bench test at a static preload of ~ 5mN. THD = 1.7%.
(PDF)

S6 Fig Displacement output of the Codacs actuator driven by an electrical 1 kHz sine wave input signal in the 3^rd^ bench test at a static preload of ~ 5mN. THD = 4.2%.
(PDF)

S7 Fig RW displacement responses to sound of TBs used for experiments (N = 10). (PDF)

S8 Fig RW displacement responses to Codacs stimulation with the Bell prosthesis at the Stapes head for nominally 1 V_rms_ actuator input voltage. (PDF)

S9 Fig SFP displacement responses to Codacs stimulation with the Bell prosthesis at the Stapes head for nominally 1 V_rms_ actuator input voltage. (PDF)

S10 Fig RW displacement responses to Codacs stimulation with the Omega/Aerial prosthesis at the Stapes head for nominally 1 V_rms_ actuator input voltage. (PDF)

S11 Fig SFP displacement responses to Codacs stimulation of the RW membrane for nominally 1 V_rms_ actuator input voltage. (PDF)

S12 Fig RW displacement responses to Codacs stimulation through a SFP fenestration with a K-piston for nominally 1 V_rms_ actuator input voltage. (PDF)
