# Supplementary figures and images for: The Codacs™ Direct Acoustic Cochlear Implant Actuator: Exploring Alternative Stimulation Sites and Their Stimulation Efficiency
Source: PLoS One. 2015 Mar 18;10(3):e0119601. doi: 10.1371/journal.pone.0119601 (PMC4364953; doi:10.1371/journal.pone.0119601)

1st Bench Test – Actuator Output at ~ 5 mN

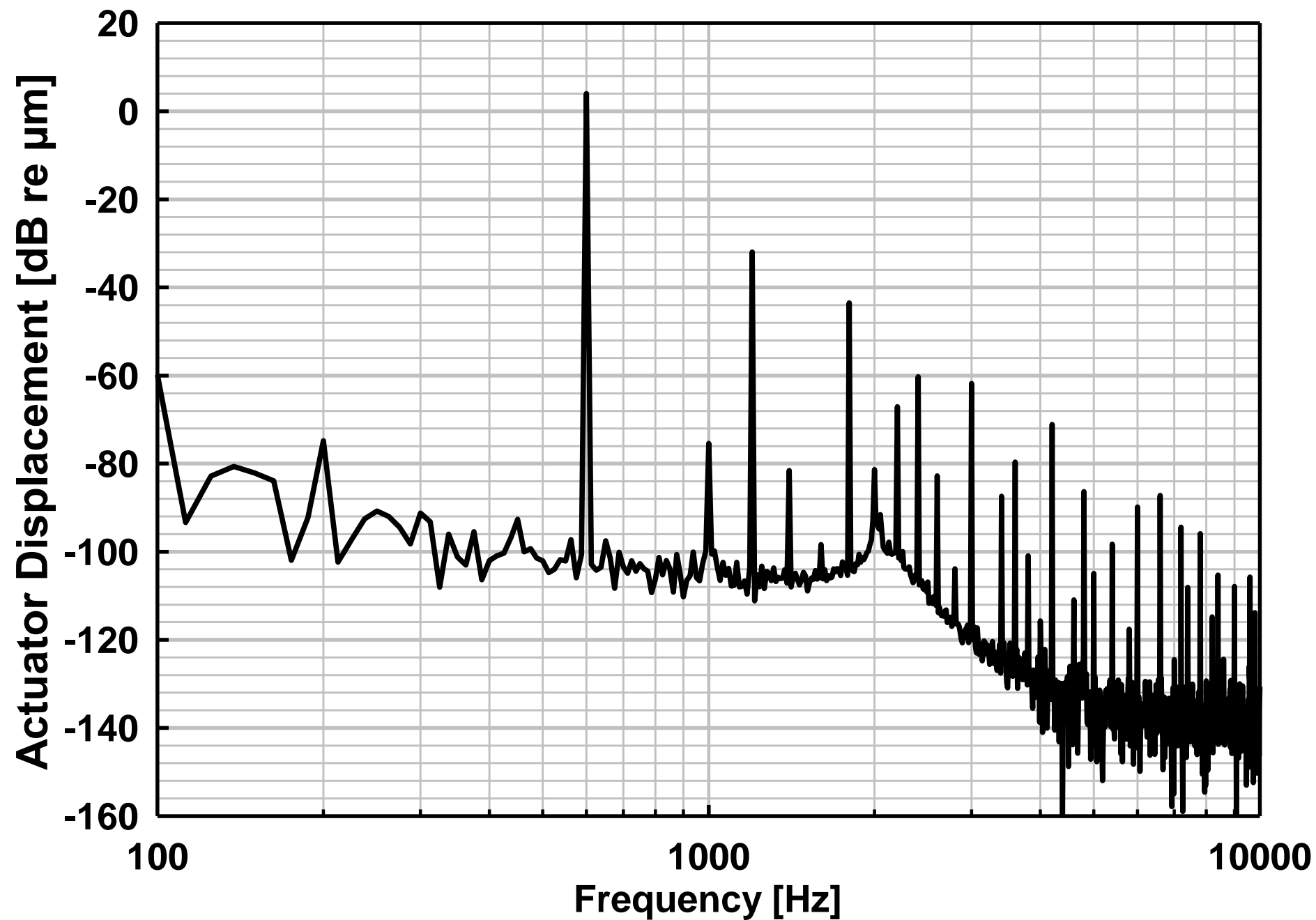

Supplement: S1 Fig — THD = 1.7% (PDF) [file pone.0119601.s001.pdf]

1st Bench Test – Actuator Output at ~ 5 mN

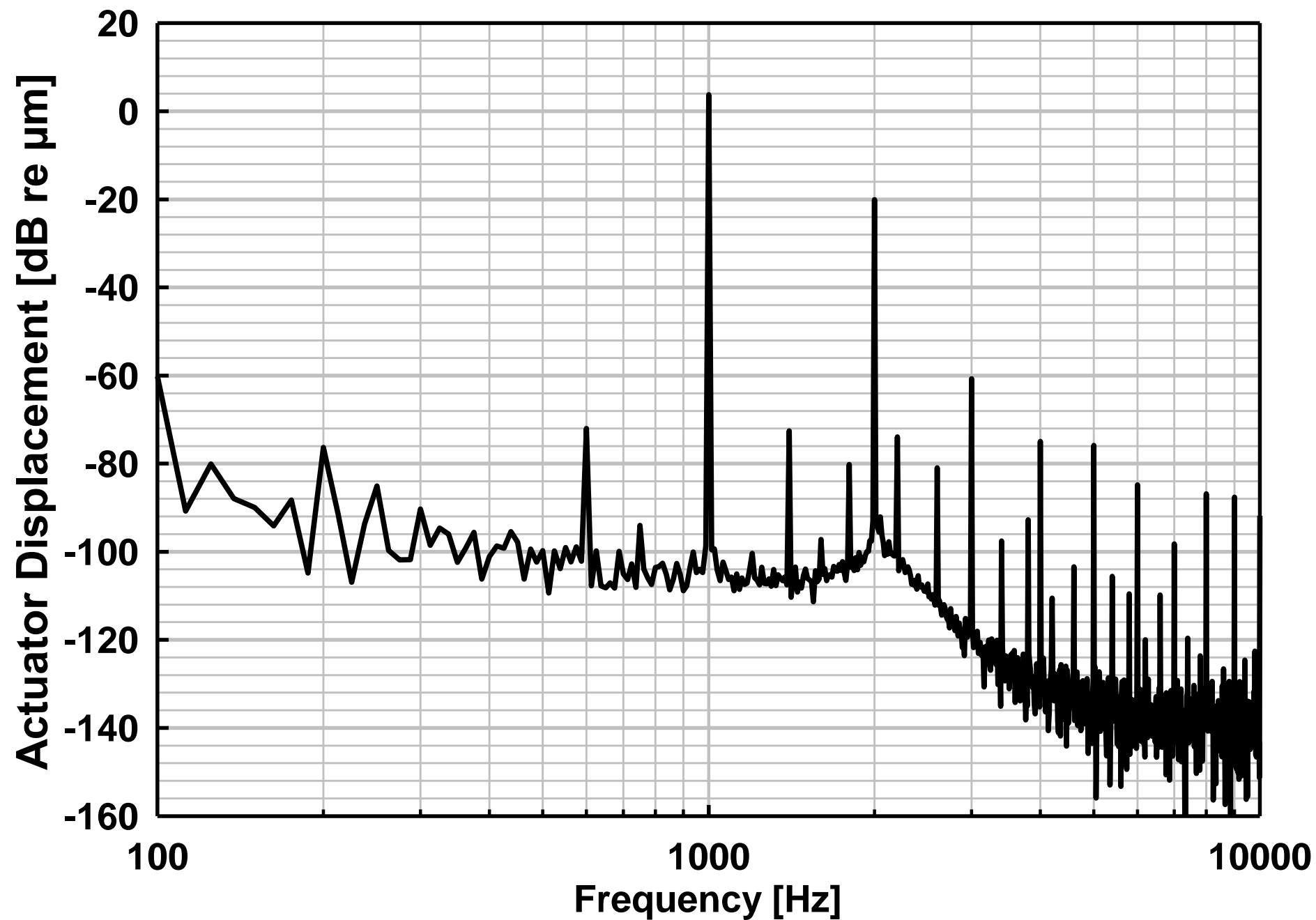

Supplement: S2 Fig — THD = 6.5%. (PDF) [file pone.0119601.s002.pdf]

2nd Bench Test – Actuator Output at ~ 5 mN

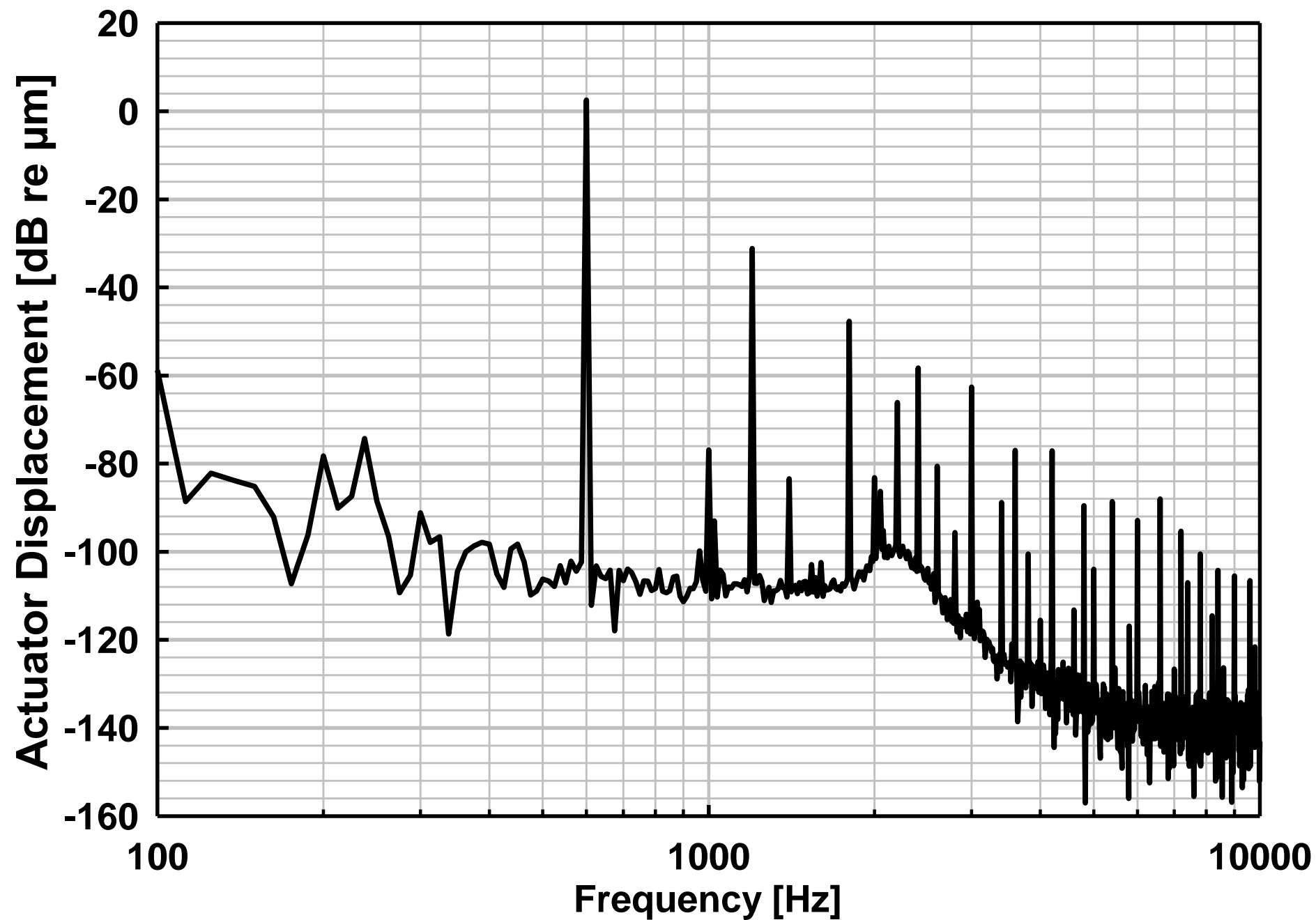

Supplement: S3 Fig — THD = 2.1%. (PDF) [file pone.0119601.s003.pdf]

**2nd Bench Test – Actuator Output at ~ 5 mN**

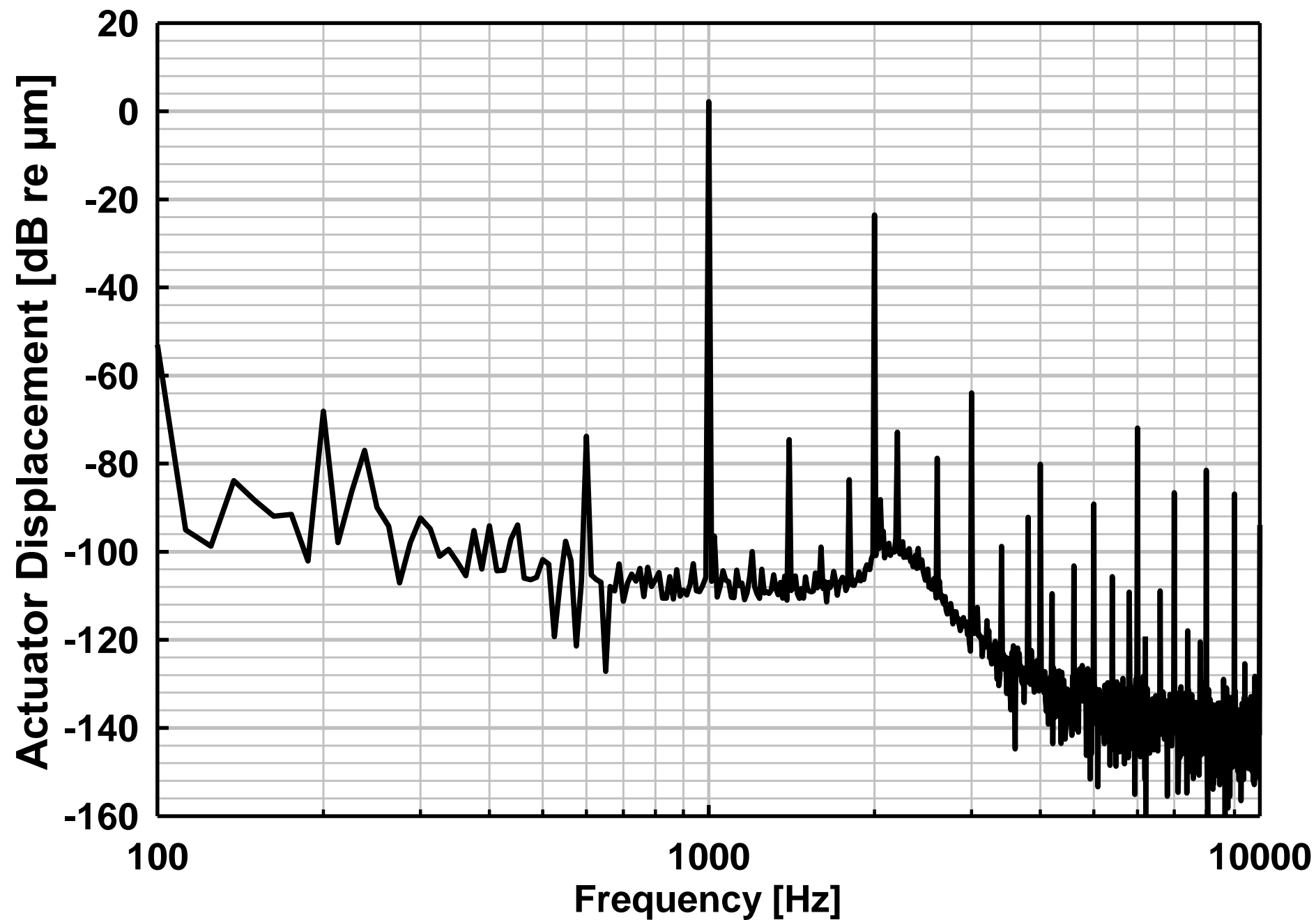

Supplement: S4 Fig — THD = 5.2%. (PDF) [file pone.0119601.s004.pdf]

3rd Bench Test – Actuator Output at ~ 5 mN

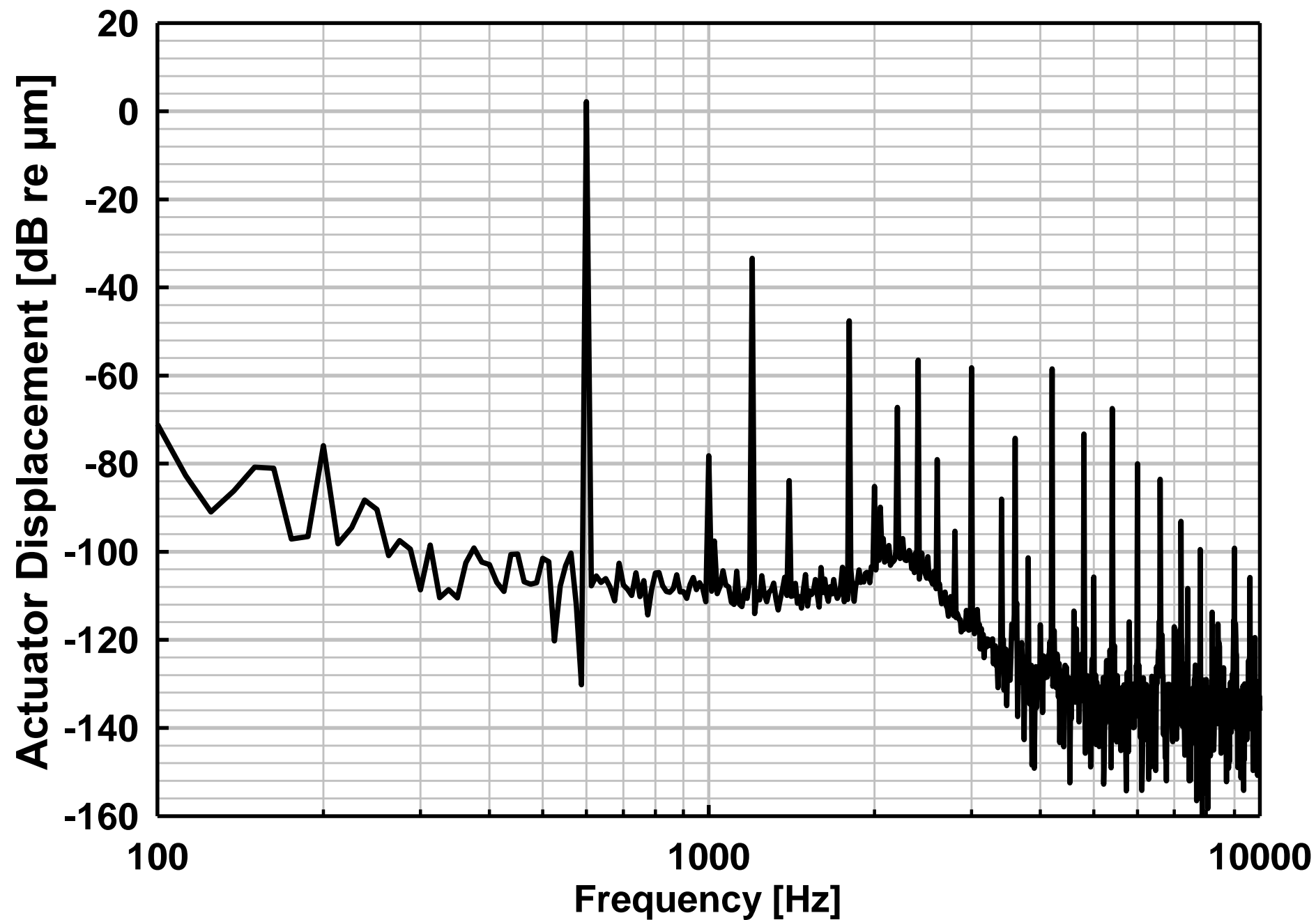

Supplement: S5 Fig — THD = 1.7%. (PDF) [file pone.0119601.s005.pdf]

1st Bench Test – Actuator Output at ~ 5 mN

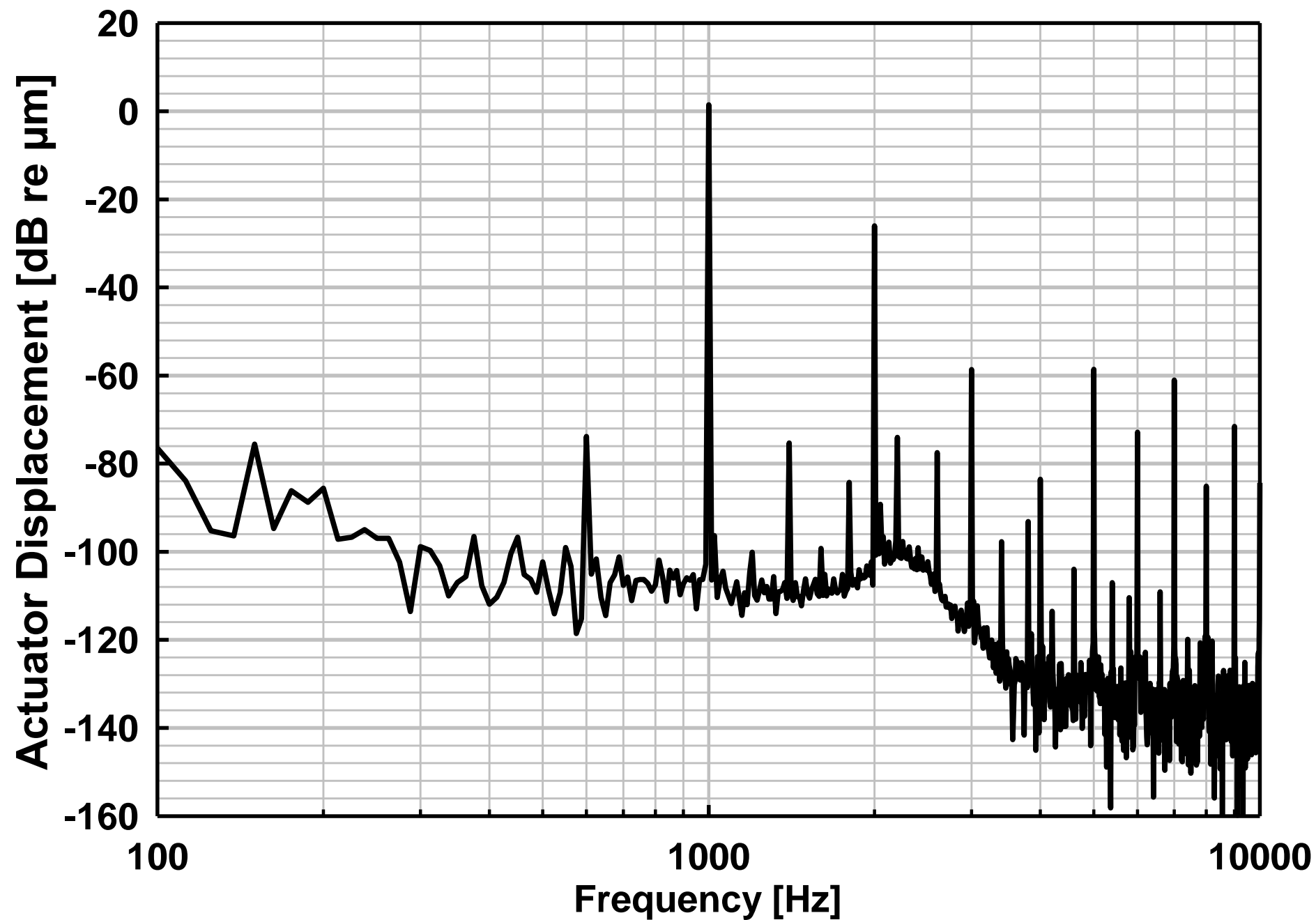

Supplement: S6 Fig — THD = 4.2%. (PDF) [file pone.0119601.s006.pdf]

# RW Displacement Response to Sound

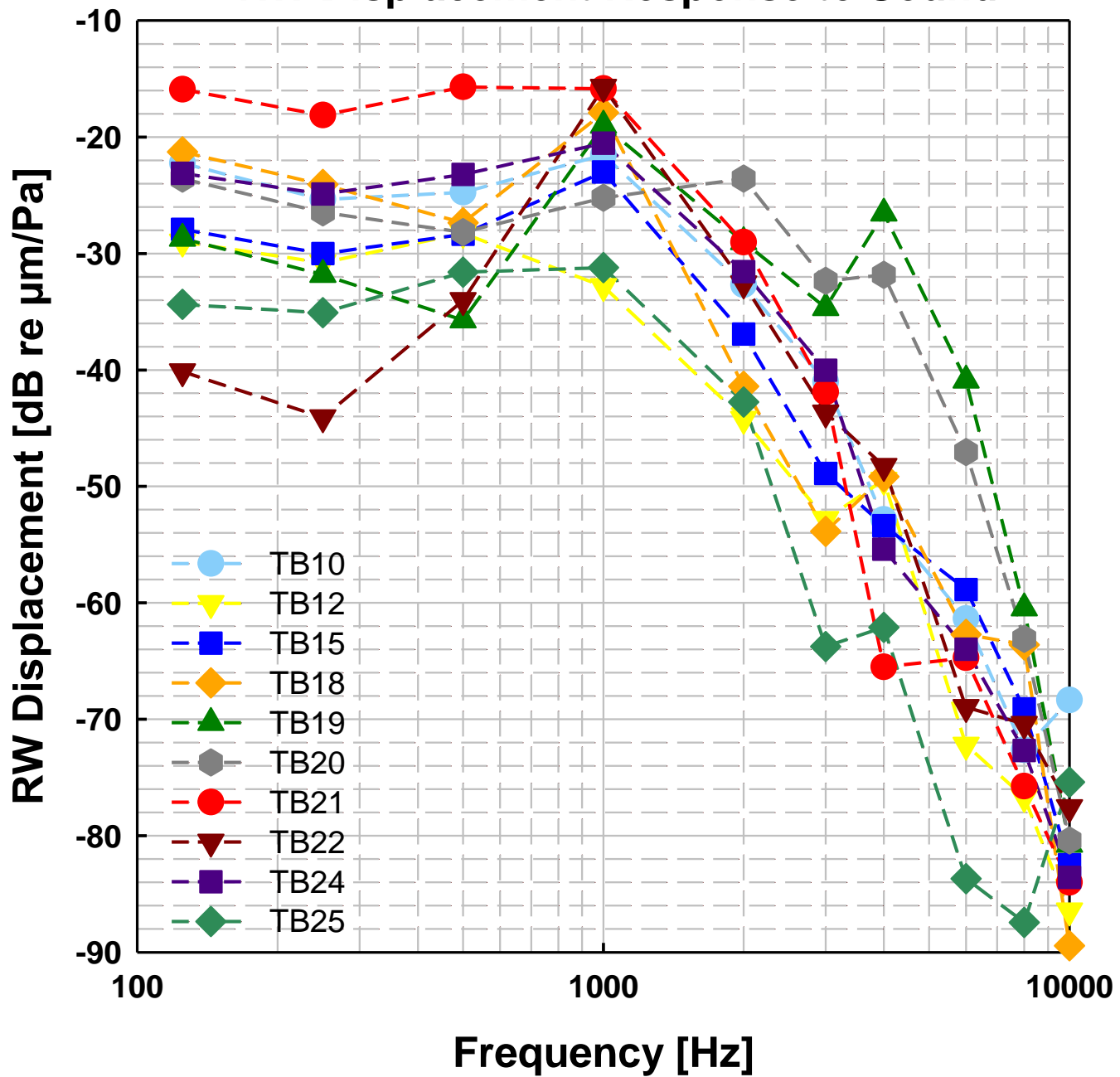

Supplement: S7 Fig — (PDF) [file pone.0119601.s007.pdf]

# Bell Prosthesis Stimulation Mode

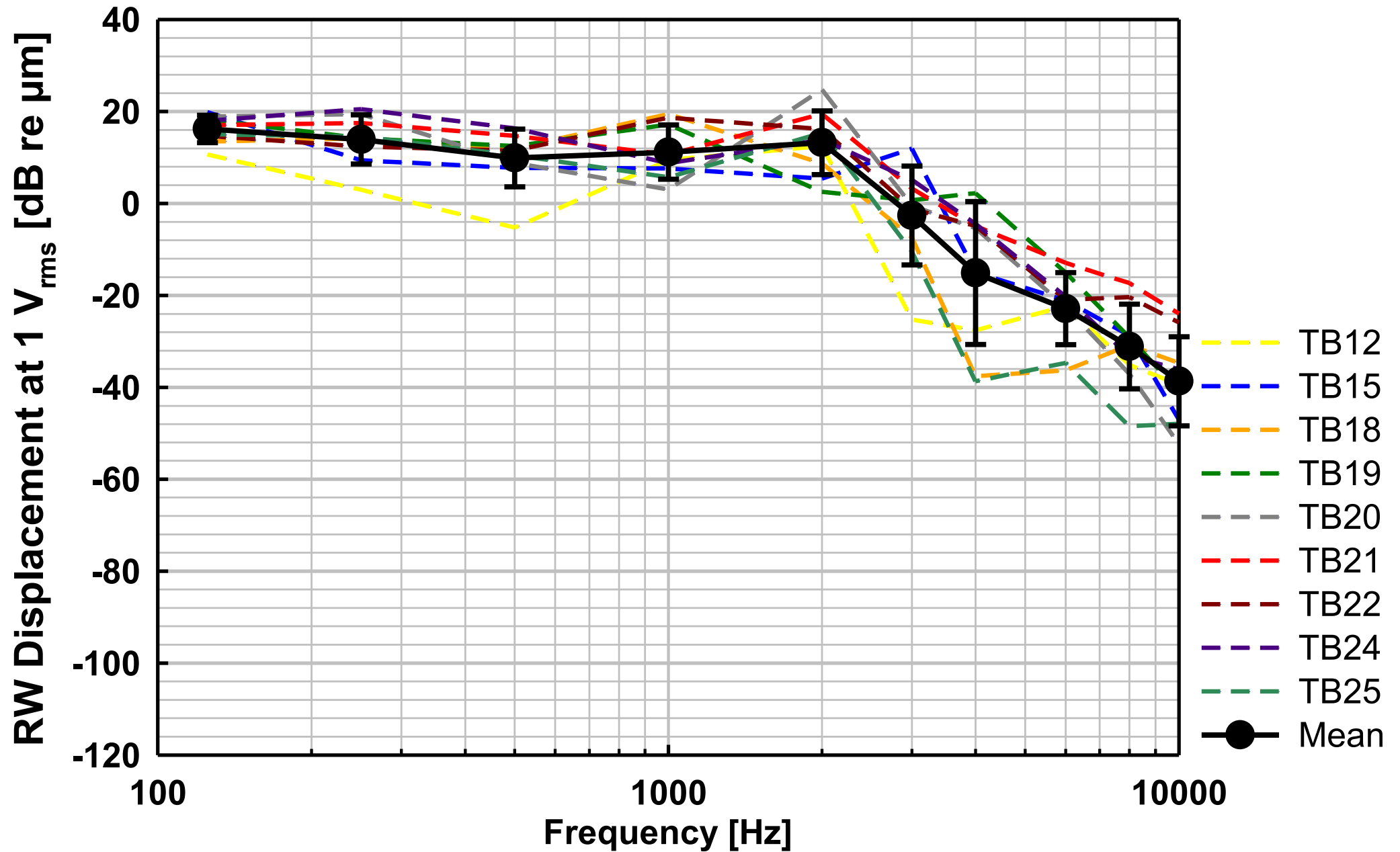

Supplement: S8 Fig — (PDF) [file pone.0119601.s008.pdf]

# Bell Prosthesis Stimulation Mode

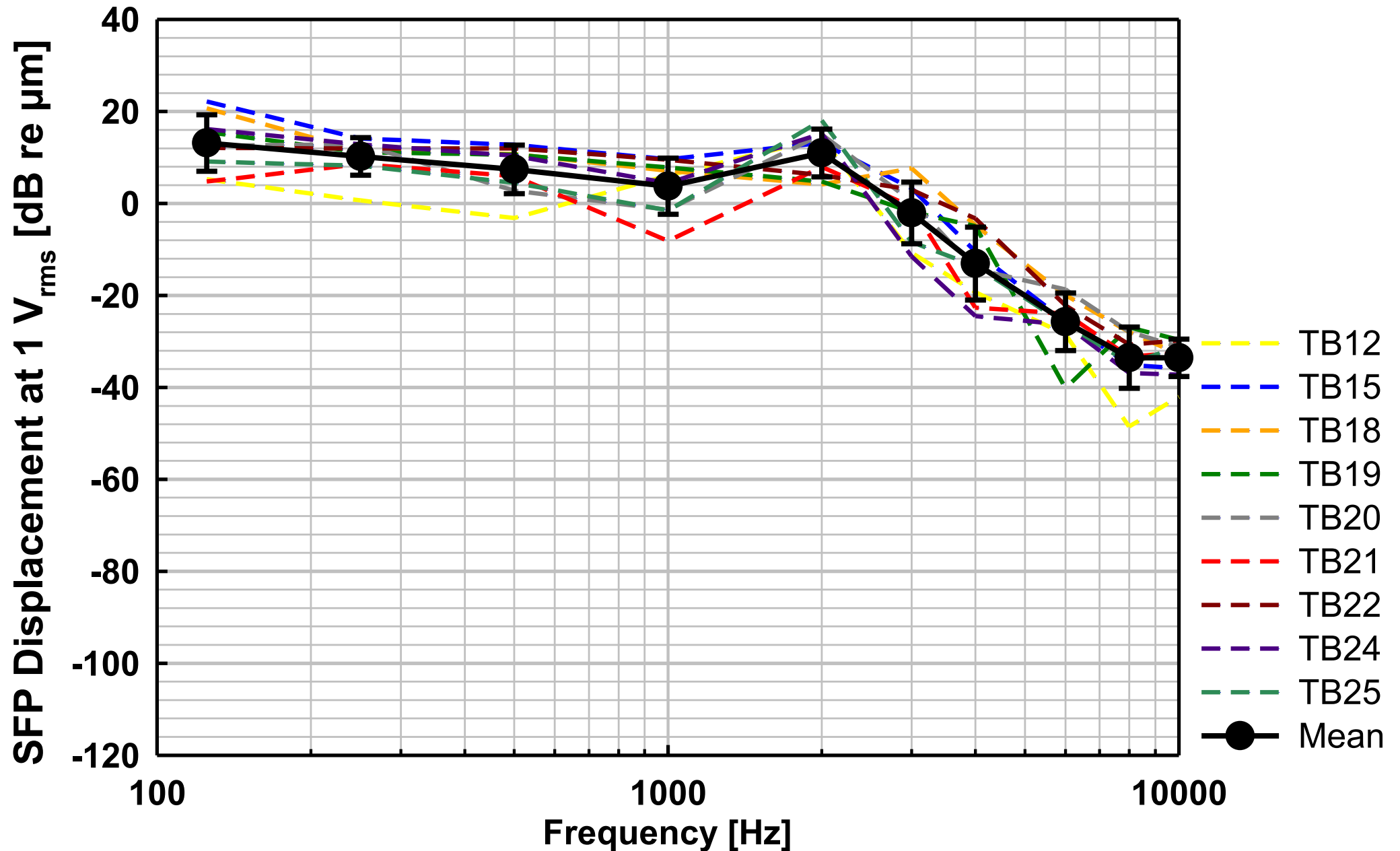

Supplement: S9 Fig — (PDF) [file pone.0119601.s009.pdf]

# Omega/Aerial Prosthesis Stimulation Mode

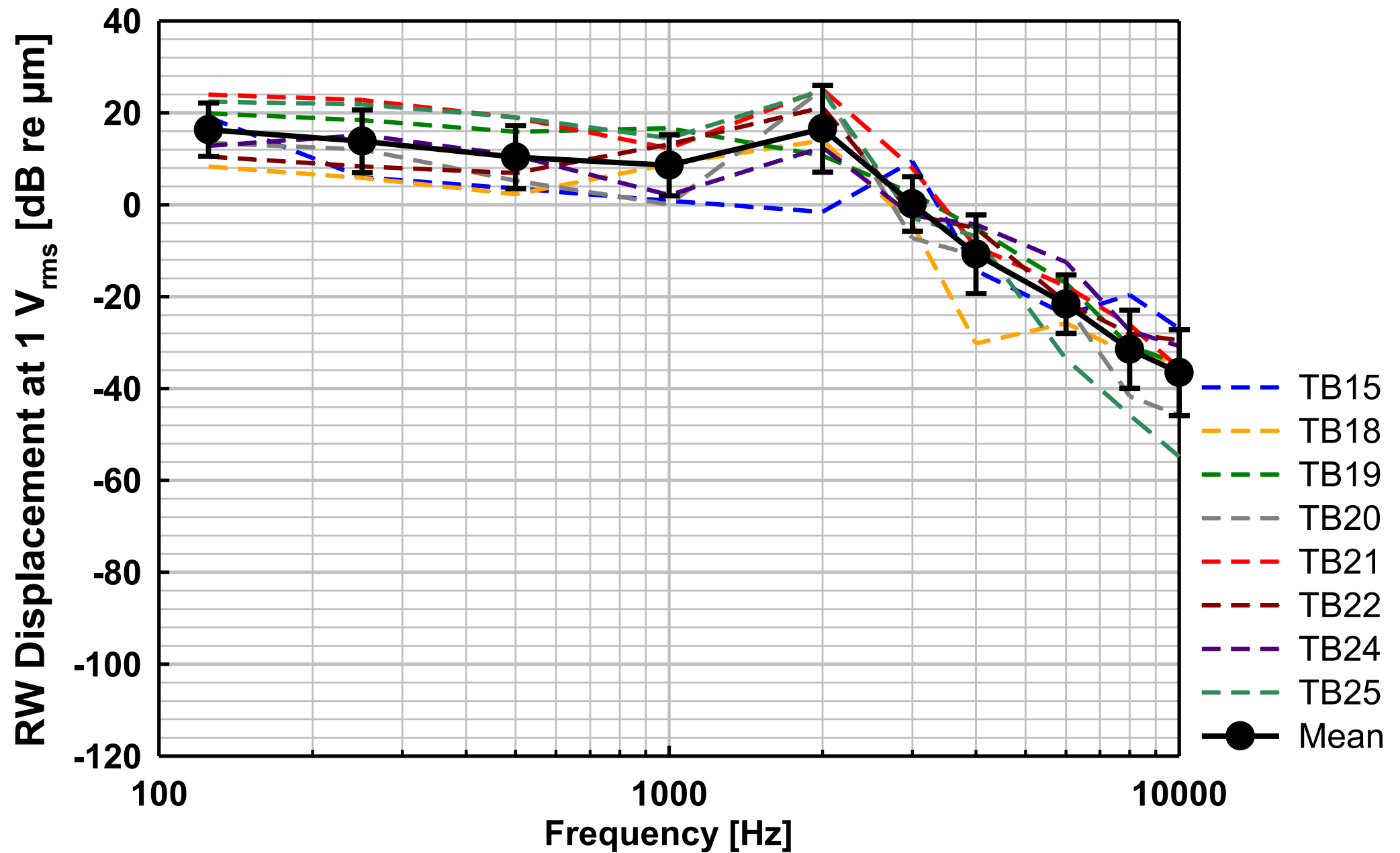

Supplement: S10 Fig — (PDF) [file pone.0119601.s010.pdf]

# Round Window Stimulation Mode

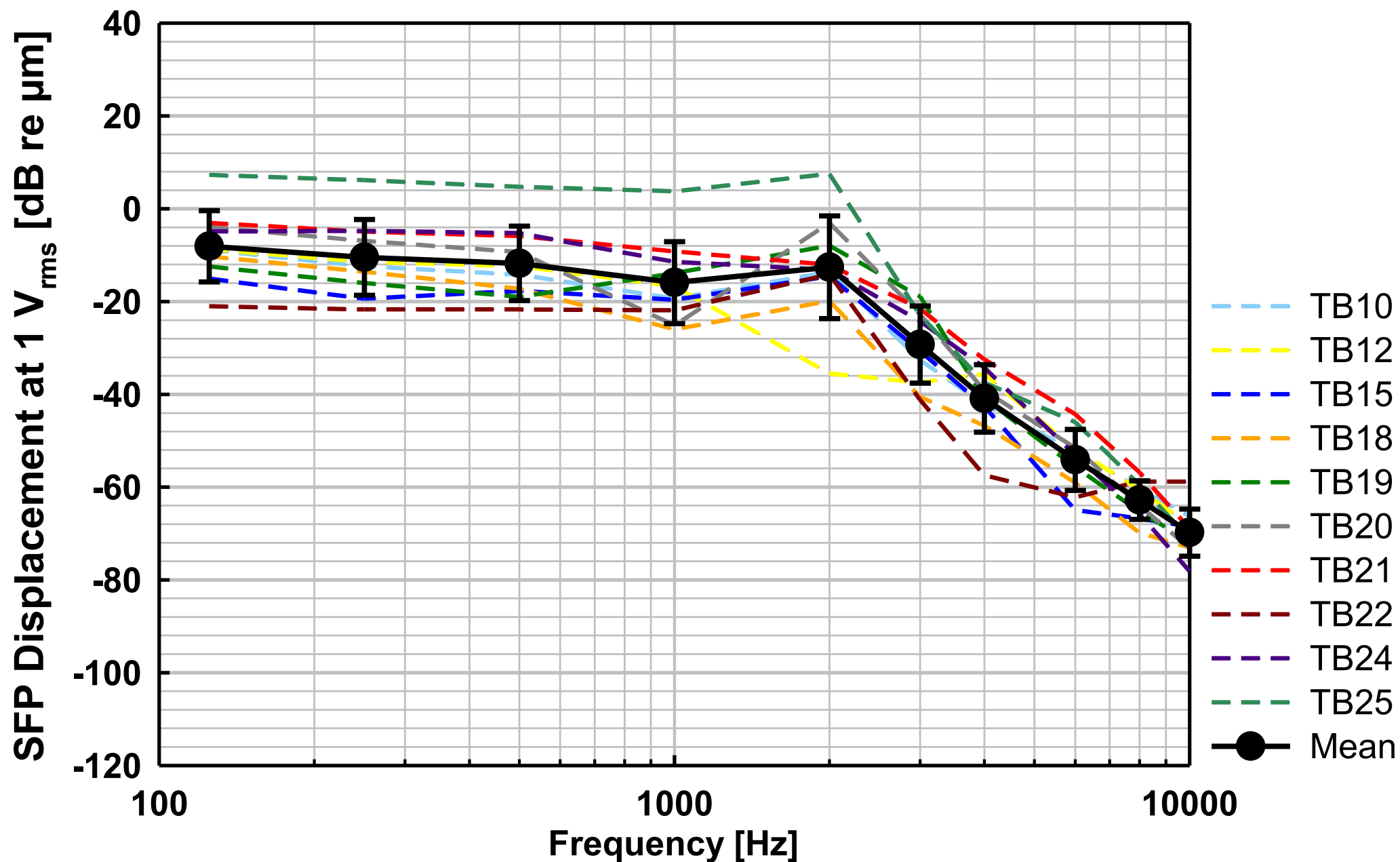

Supplement: S11 Fig — (PDF) [file pone.0119601.s011.pdf]

# K-Piston Prosthesis Stimulation Mode

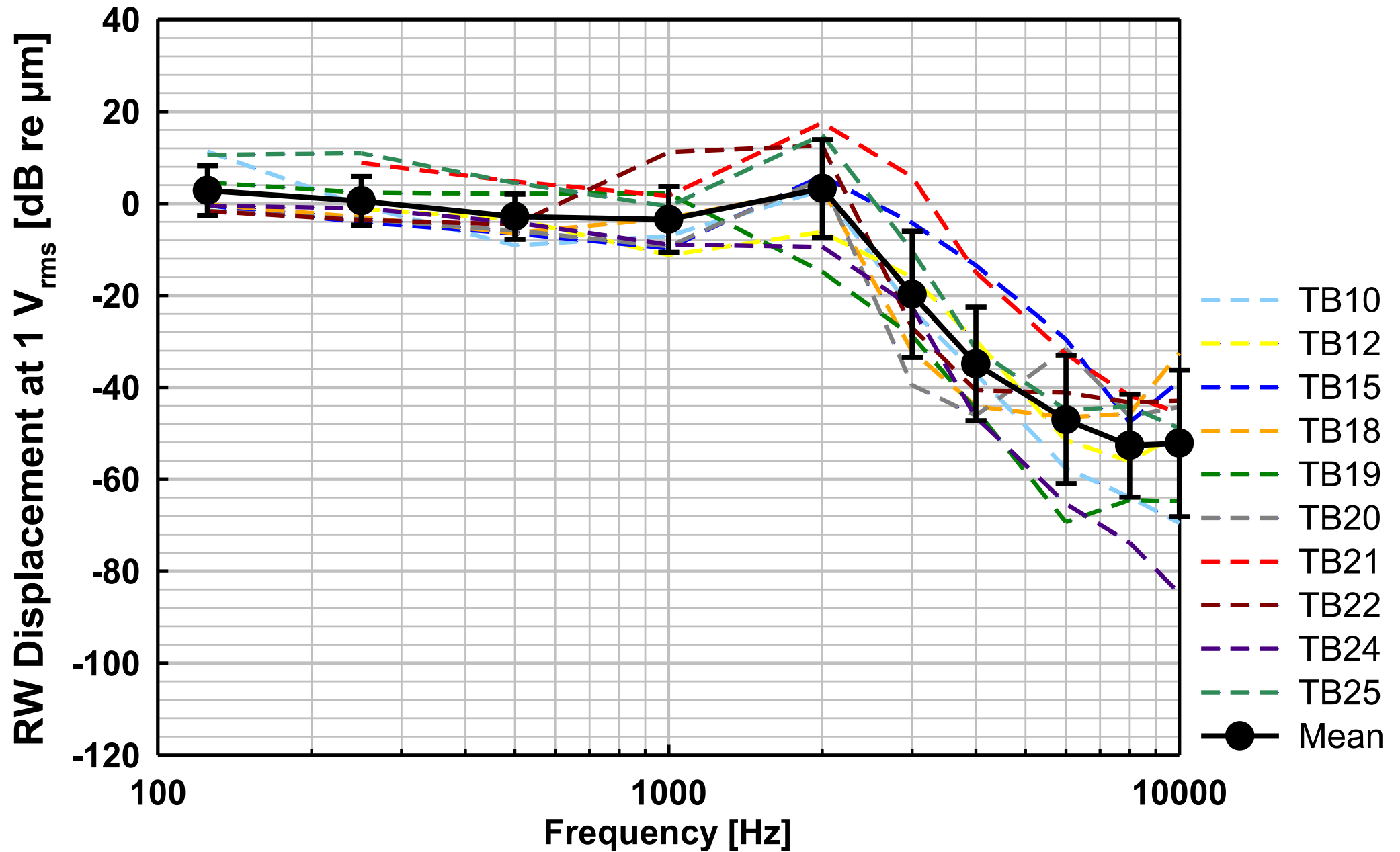

Supplement: S12 Fig — (PDF) [file pone.0119601.s012.pdf]
